# Fragile X mental retardation protein in intrahepatic cholangiocarcinoma: regulating the cancer cell behavior plasticity at the leading edge

**DOI:** 10.1038/s41388-021-01824-3

**Published:** 2021-05-20

**Authors:** Simone Carotti, Maria Zingariello, Maria Francesconi, Laura D’Andrea, M. Ujue Latasa, Leticia Colyn, Maite G. Fernandez-Barrena, Rocco Simone Flammia, Mario Falchi, Daniela Righi, Giorgia Pedini, Francesco Pantano, Claudia Bagni, Giuseppe Perrone, Rosa Alba Rana, Matias A. Avila, Sergio Morini, Francesca Zalfa

**Affiliations:** 1 Research Unit of Microscopic and Ultrastructural Anatomy, Department of Medicine, Campus Bio-Medico University, Rome, Italy; 2 Predictive Molecular Diagnostic Unit, Department of Pathology, Campus Bio-Medico University Hospital, Rome, Italy; 3 Hepatology Program, Center for Applied Medical Research (CIMA), University of Navarra and IdiSNA, Pamplona, Spain; 4 Centro de Investigación Biomédica en Red, Enfermedades Hepáticas y Digestivas (CIBERehd), Madrid, Spain; 5 National AIDS Center, Istituto Superiore di Sanità, Rome, Italy; 6 Department of Biomedicine and Prevention, University of Rome Tor Vergata, Rome, Italy; 7 Medical Oncology Department, Campus Bio-Medico University, Rome, Italy; 8 Department of Fundamental Neurosciences, University of Lausanne, Lausanne, Switzerland; 9 Research Unit of Pathology, Campus Bio-Medico University, Rome, Italy; 10 Medicine and Aging Science Department, University G. D’Annunzio, Chieti-Pescara, Italy

**Keywords:** Liver cancer, Mechanisms of disease

## Abstract

Intrahepatic cholangiocarcinoma (iCCA) is a rare malignancy of the intrahepatic biliary tract with a very poor prognosis. Although some clinicopathological parameters can be prognostic factors for iCCA, the molecular prognostic markers and potential mechanisms of iCCA have not been well investigated. Here, we report that the Fragile X mental retardation protein (FMRP), a RNA binding protein functionally absent in patients with the Fragile X syndrome (FXS) and also involved in several types of cancers, is overexpressed in human iCCA and its expression is significantly increased in iCCA metastatic tissues. The silencing of FMRP in metastatic iCCA cell lines affects cell migration and invasion, suggesting a role of FMRP in iCCA progression. Moreover, we show evidence that FMRP is localized at the invasive front of human iCCA neoplastic nests and in pseudopodia and invadopodia protrusions of migrating and invading iCCA cancer cells. Here FMRP binds several mRNAs encoding key proteins involved in the formation and/or function of these protrusions. In particular, we find that FMRP binds to and regulates the expression of Cortactin, a critical regulator of invadopodia formation. Altogether, our findings suggest that FMRP could promote cell invasiveness modulating membrane plasticity and invadopodia formation at the leading edges of invading iCCA cells.

## Introduction

Intrahepatic cholangiocarcinoma (iCCA) is a primary cancer of the bile ducts arising from the malignant transformation of biliary epithelial cells and is characterized by an aggressive behavior with early lymphatic and metastatic spread and very poor prognosis [1]. iCCA is relatively uncommon, but its incidence is on the increase [2]. Up to date, surgical resection of iCCA represents the only potentially curative therapeutic option, but most of the patients are ineligible for curative surgery because of the presence of metastases at the time of diagnosis [3]. Therefore, the identification of molecular biomarkers, as well as novel promising molecules for targeted therapy development is of primary importance in iCCA clinical management.

Here, we investigated the role of the Fragile X Mental Retardation Protein (FMRP) in iCCA. FMRP is a selective RNA binding protein whose functional absence causes the Fragile X Syndrome (FXS), an X-linked neurological disorder that represents the most common form of inherited intellectual disability in humans [4, 5]. FMRP has been largely studied and characterized in brain and, in particular, in neurons where its functional absence causes dendritic spine dysmorphogenesis [6–8]. The FMRP mRNA target-transcriptome was characterized in neurons [9–12], and an IPA analysis of FMRP-regulated mRNAs has shown that these targets are mainly involved in cytoskeleton remodeling and cell-to-cell signaling and interaction [6], mechanisms also involved in cancer progression. Indeed, it was recently shown that FMRP is also involved in progression of breast cancer [13], astrocytoma [14], malignant pleural mesothelioma [15], melanoma [16], and colon cancer [17]. Of note, in melanoma cells we identified the FMRP-regulated transcriptome that further confirmed its implication in the invasion of the tumor [16]. In agreement with these data, a decreased risk of cancer [13, 18], as well as an unusual reduction of cancer invasiveness [19] in individuals with FXS, have been observed.

Finally, some evidence suggests an involvement of FMRP in hepatic and pancreatic cancers [20–23]. In particular, *FMR1* gene, encoding for FMRP, is overexpressed in both pre-neoplastic and neoplastic stages of HCC [20, 21] and was identified as a metastasis-related gene in an HCC cell line derived from lung metastatic lesions [22], as well as a downstream effector of NMDAR signaling pathway that influences invasive tumor growth in a mouse model of pancreatic neuroendocrine tumor (PanNET) [23].

Despite this evidence in liver and pancreatic cancers, the role of FMRP in cholangiocarcinoma has never been investigated before. Here we show that FMRP is overexpressed in a selected cohort of human iCCA and in particular the highest levels of expression are found in metastatic tissues. Furthermore, in metastatic iCCA cell lines, the silencing or the overexpression of FMRP influences cellular migration and invasion, suggesting a role of FMRP in iCCA progression. At the cellular level, by confocal microscopy, immunogold electron microscopy (EM) and time-lapse live imaging, FMRP was found in cytoplasmic RNA granules accumulating in the protrusions of the plasma membrane involved in migration and invasion, such as pseudopodia and invadopodia, respectively. Of note, the reduction of FMRP expression decreases the ability of iCCA cells to form invadopodia protrusions and to degrade extracellular matrix in vitro. Finally, using a co-immunoprecipitation/NanoString nCounter^®^ approach we demonstrated that FMRP is able to bind several mRNAs encoding key proteins required for pseudopodia and invadopodia formation and function. In particular, FMRP is able to bind *Cortactin* (*CTTN)* mRNA and to regulate the expression of Cortactin, a critical regulator of invadopodia formation. Our data strongly suggest a role for FMRP in regulating mRNA metabolism at the leading edges of iCCA cells.

## Results

### FMRP is overexpressed in human iCCA and its levels correlate with the metastatic phenotype

**Fig. 1:**
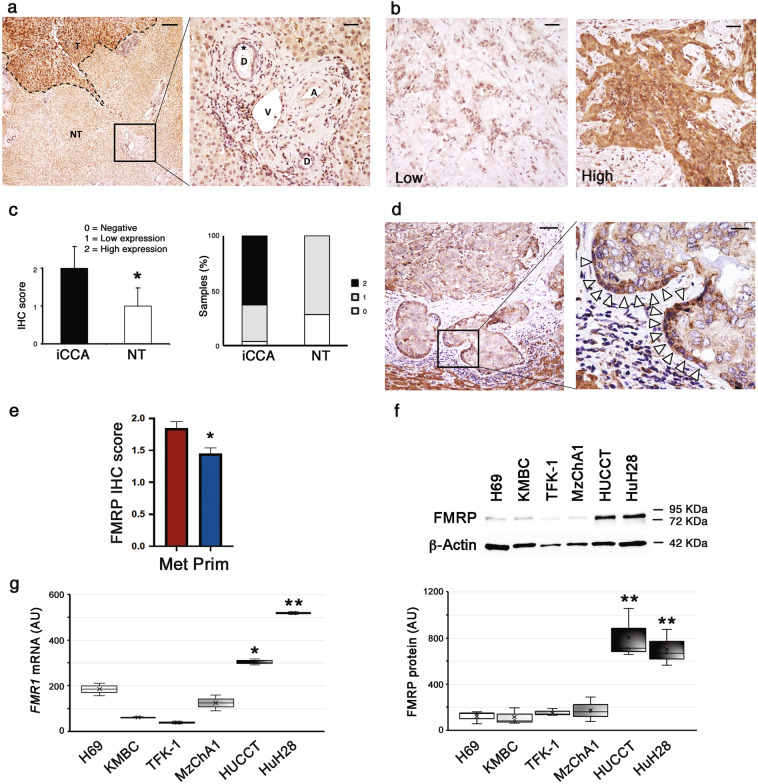
FMRP is overexpressed in human iCCA. **a** FMRP immunohistochemical expression in non tumoral hepatic tissues (NT region) and in iCCA (T region). In left panel, FMRP expression is higher in tumoral tissue (T, dashed line) compared to non tumoral liver (NT). The box in left panel indicates the high power field in right panel. In the surrounding non tumoral liver, cholangiocytes of biliary ducts (asterisk) in portal tracts do not show a significant FMRP expression (D = biliary ducts, V = portal vein, A = hepatic artery). Calibration bars: 200 µm (left panel), 40 µm (right panel). **b** FMRP immunohistochemical expression in human iCCA samples was observed with low (left panel) or high (right panel) expression levels. Calibration bars: 20 µm. **c** FMRP expression was evaluated by IHC in 48 human iCCA samples and seven non-tumoral hepatic tissues (NT). Each sample was scored by using a semi-automated method and the IHC score of iCCA and NT tissues was reported in the left histogram. The columns represent median values and the bars, standard deviations (*p < 0.05; Mann–Witney test). In the right histogram the percentage of samples (iCCA or NT) with 0, 1, or 2 scores was plotted. **d** A representative IHC of an iCCA sample with low FMRP expression, showing the heterogeneous staining of FMRP in the neoplastic nests. The box in the left panel indicates the high-power field in the right panel. Arrowheads indicate FMRP immunopositivity at the boundaries of neoplastic nests. Calibration bars: 100 µm (left panel), 20 µm (right panel). **e** FMRP immunohistochemical expression in human iCCA cohort was compared with metastatic (Met) or primary (Prim) nature of tumor samples. The columns represent median values and the bars, standard errors (*p < 0.05; Mann–Witney test). **f** Representative western blotting for FMRP and β-Actin in intrahepatic normal human biliary epithelial cell line H69, in three eCCA cell lines (KMBC, TFK-1, and MzChA1) and in two iCCA cell lines (HuCCT and HuH28) protein extracts (10 μg). In the boxplot the densitometric quantification of FMRP bands (normalised for β-Actin bands) in three independent western blotting experiments. The boxes represent quartiles, the ×, the median and the whiskers, the variability beyond the upper and lower quartiles (**p < 0.01; Student’s *t* test). **g** *FMR1* mRNA expression levels were evaluated by RT-qPCR in the same cell lines of panel (**f**). In the boxplot, the boxes represent quartiles, the ×, the median and the whiskers, the variability beyond the upper and lower quartiles (*p < 0.05 and **p < 0.01; Student’s *t* test).

The expression and the intra-tissue distribution of FMRP were evaluated by immunohistochemistry (IHC) in a cohort of iCCAs (*n* = 48) and non-tumoral hepatic tissues (*n* = 7). In non-tumoral hepatic tissues, cholangiocytes of bile ducts were negative (IHC score = 0) or showed low FMRP expression (IHC score = 1), while a clear immunopositivity was found in the surrounding hepatocytes (Fig. 1a, left panel, NT region and right panel, see asterisk). However, in iCCA FMRP was detected (Fig. 1a, left panel, T region) with an expression level ranging from low (IHC score = 1) to high (IHC score = 2) (Fig. 1b, c), and with an overall significant overexpression in the tumor tissues, compared to non-neoplastic bile ducts (Fig. 1c, left panel). In particular, the FMRP immunopositivity was mainly found at the margins of the neoplastic glands or nests, close to the neighboring stromal tissue (tumor-stromal interface) (Fig. 1d, see arrowheads).

Interestingly, a comparative analysis with clinic-pathological parameters highlighted a significant correlation between FMRP expression and metastatic nature of iCCA tissues, suggesting a role for FMRP in iCCA progression and metastatization (Fig. 1e).

Conversely, in a cohort of extrahepatic cholangiocarcinomas (eCCA) (*n* = 17) FMRP was not significantly overexpressed compared to non-tumoral hepatic tissues (Supplementary Fig. 1a), suggesting that the different FMRP expression might be due to the different nosological entity of the iCCA respect to eCCA.

Next, we evaluated the FMRP expression levels in an immortalized intrahepatic normal human biliary epithelial cell line (H69) [24], in three human metastatic eCCA cell lines (KMBC, TFK-1 and MzChA1) and in two human metastatic iCCA cell lines (HuCCT and HuH28) [25]. FMRP and *FMR1* mRNA were significantly overexpressed in the two iCCA cell lines compared to both non-transformed biliary epithelial cells and eCCA cell lines (Fig. 1f for proteins and Fig. 1g for mRNAs), in agreement with the IHC data.

### FMRP is localized at the leading edges of iCCA cells

**Fig. 2:**
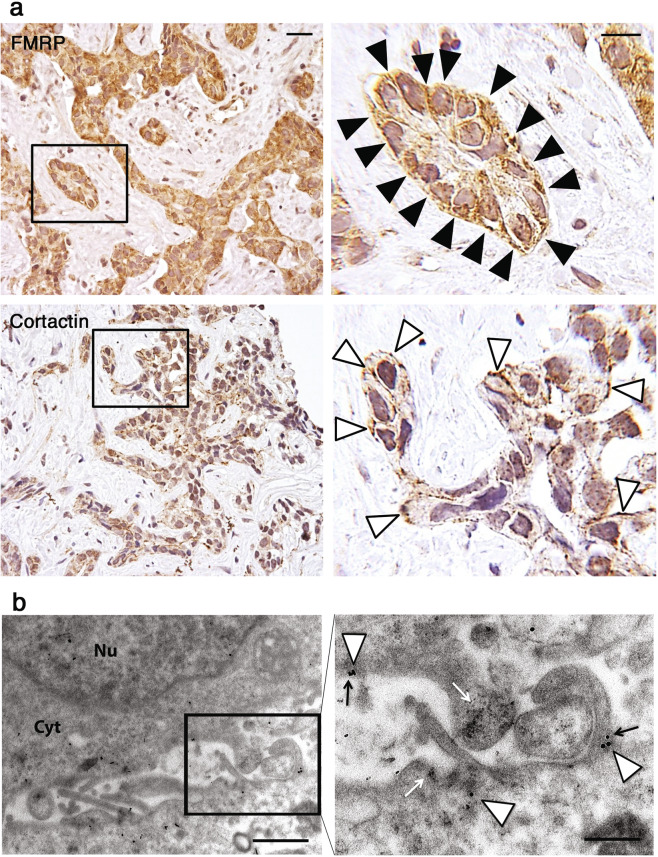
Immunohistochemical and ultrastructural localization of FMRP at the leading edge in iCCA cells. **a** Representative images of a IHC for FMRP in an iCCA sample, showing FMRP staining mainly localized beneath plasma membrane of iCCA cells, with a strong enhancement of signal towards the basal lamina (upper panels, black arrowhead). Cortactin staining exhibits a similar intracellular pattern (lower panels, white arrowhead). Calibration bars 20 µm. Original magnification 20× (left panels), high power fields 100× (right panels). **b** Ultrathin sections of HuCCT cellular pellets were subjected to immunogold electron microscopy with specific FMRP and Cortactin antibodies. The box in left panel indicates the high-power field in right panel. 25 nm gold particles (FMRP, white arrowheads) and 15 nm gold particles (Cortactin, black arrows) colocalized at the base of membrane protrusions of HuCCT cells. The white arrows indicate ribosomes densities. *Nu* Nucleus, *Cyt* Cytoplasm. Left panel: original magnification ×18,500. Scale bar: 1 μm. Right panel: original magnification ×37,000. Scale bar: 500 nm.

We also investigated the intracellular distribution of FMRP in iCCA cells. In human iCCA tissues IHC revealed a dotted positivity pattern linearly gathered under plasma membrane (Fig. 2a, top panels, see black arrowheads), very similar to the pattern of Cortactin protein (Fig. 2a, bottom panels, see white arrowheads), a key regulator of actin cytoskeleton dynamics and cell shape at the leading edges [26]. Using immunocytochemistry EM we also observed FMRP gold particles localized inside and at the base of membrane protrusions at the leading edges of both HuCCT (Fig. 2b, white arrowheads) and HuH28 (Supplementary Fig. 1b, white arrowheads) cells, sometimes near Cortactin gold particles (Fig. 2b, black arrows) and polyribosomes aggregates (Fig. 2b, white arrows).

### FMRP is localized in pseudopodia protrusions at the leading edge of migrating iCCA cells

**Fig. 3:**
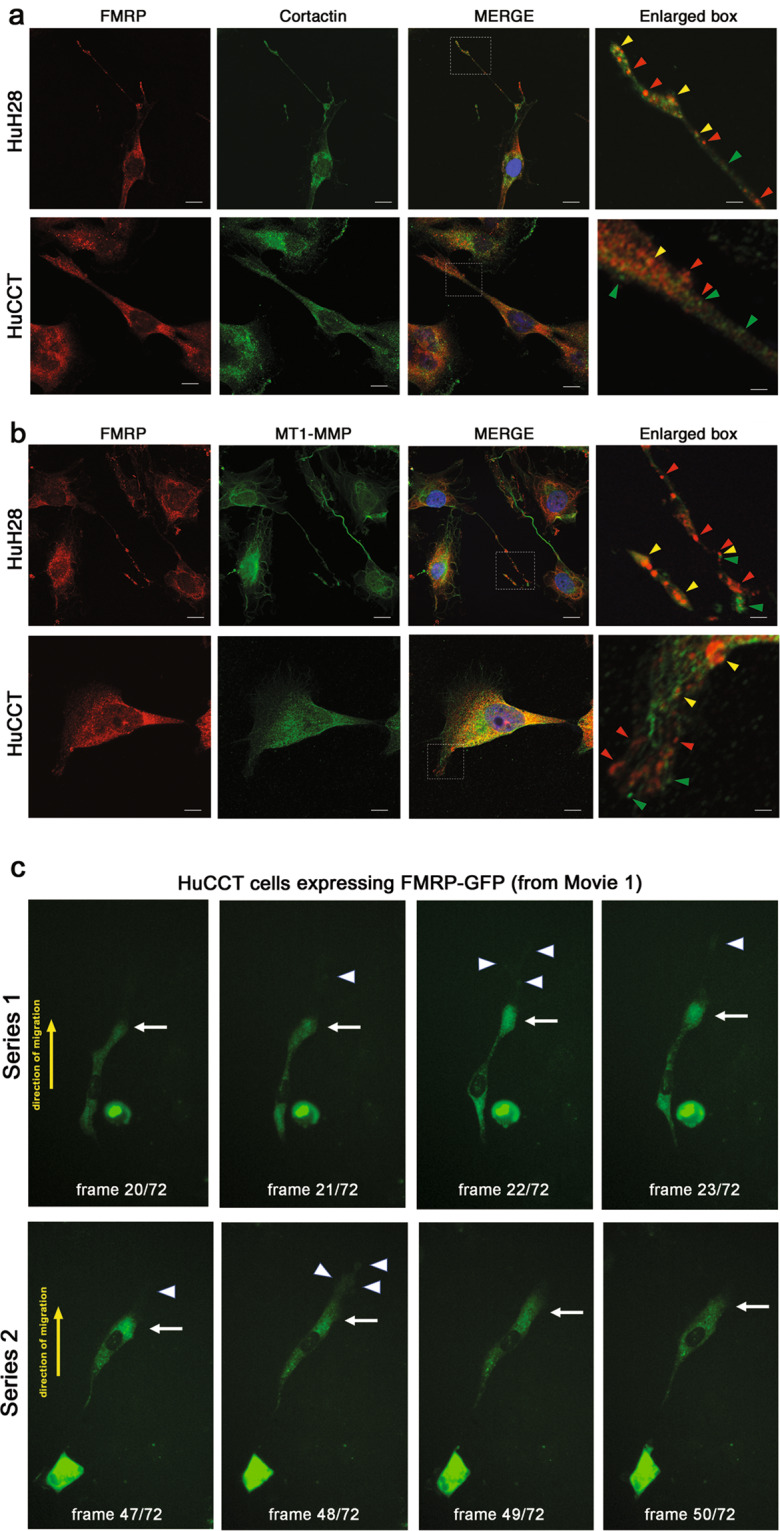
FMRP localizes in cytoplasmic granules inside Cortactin and MT1-MMP –enriched cell protrusions. **a** HuH28 or HuCCT cells were grown on fibronectin-coated chamber slides for 48 h and then IF was performed. Cells were stained with FMRP (red), Cortactin (green) and nuclei were stained with DAPI (blue). In the last right panels, enlarged images of white box in MERGE panels. Red arrowheads, FMRP granules; green arrowheads, Cortactin positive foci; yellow arrowheads, FMRP-Cortactin colocalization foci, inside cell protrusions. Bars, 20 μm in HuH28 panels; 10 μm in HuCCT panels; 4 μm in enlarged panels. **b** HuH28 or HuCCT cells were grown on fibronectin-coated chamber slides for 48 h and then IF was performed. Cells were stained with FMRP (red), MT1-MMP (green) and nuclei were stained with DAPI (blue). In the last right panels, enlarge images of MERGE panels. Red arrowheads, FMRP granules; green arrowheads, MT1-MMP positive foci; yellow arrowheads, FMRP-MT1-MMP colocalization foci, inside cell protrusions. Bars, 20 μm in HuH28 panels; 10 μm in HuCCT panels; 4 μm in enlarged panels. **c** HuCCT cells overexpressing FMRP-GFP were analysed with time-lapse fluorescence microscopy. FITC signal (green) corresponding to FMRP-GFP was acquired every 10 min for 12 h. A total of 72 acquisitions were assembled to produce a live imaging movie (see Supplementary Information). Frames 20–23 (series 1, upper panels) or 47–50 (series 2, lower panels) of Movie 1 are shown. White arrow points cellular leading edge, white arrowheads point filopodia at the leading edge and yellow arrow, the direction of cell migration. Original magnification 20×.

Invasive cell migration requires sustained forward movement of the plasma membrane at the cellular front or leading edge and is preceded by the formation of pseudopodia structures such as lamellipodia and filopodia [27]. To investigate whether FMRP–containing granules observed in iCCA cells (Fig. 2) were also present inside cell protrusions in fibronectin-migrating cells, we performed a fluorescent immunocytochemical analysis of FMRP, and simultaneously stained cell protrusions with Cortactin or MT1-MMP, two proteins enriched in both lamellipodia and invadopodia structures [28, 29]. Interestingly, FMRP granules localized in pseudopodia (filopodia or lamellipodia) protrusions at the leading edge of HuH28 and HuCCT cells (Fig. 3, red arrowheads), which also contained Cortactin (Fig. 3a, green arrowheads) and MT1-MMP (Fig. 3b, green arrowheads). Only rarely FMRP-Cortactin or FMRP-MT1-MMP colocalization foci were observed (Figs. 3a, b, yellow arrowheads), suggesting that, although present within the same cell protrusions, these proteins probably are not part of the same protein complexes. Moreover, using immunofluorescence we could detect an enrichment of FMRP granules in the distal part of the protrusions compared to proximal and medial parts (Supplementary Fig. 1c).

In addition, time-lapse live imaging of migrating HuCCT cells overexpressing FMRP-GFP, showed cytoplasmic FMRP granules moving along cell protrusions (lamellipodia and filopodia) at the leading edge (Fig. 3c and Supplementary Information Movies 1, 2), while HuCCT control cells expressing GFP showed a diffuse, uniform and not polarized signal (Supplementary Information Movie 3).

### FMRP is localized in invadopodia of iCCA cells

**Fig. 4:**
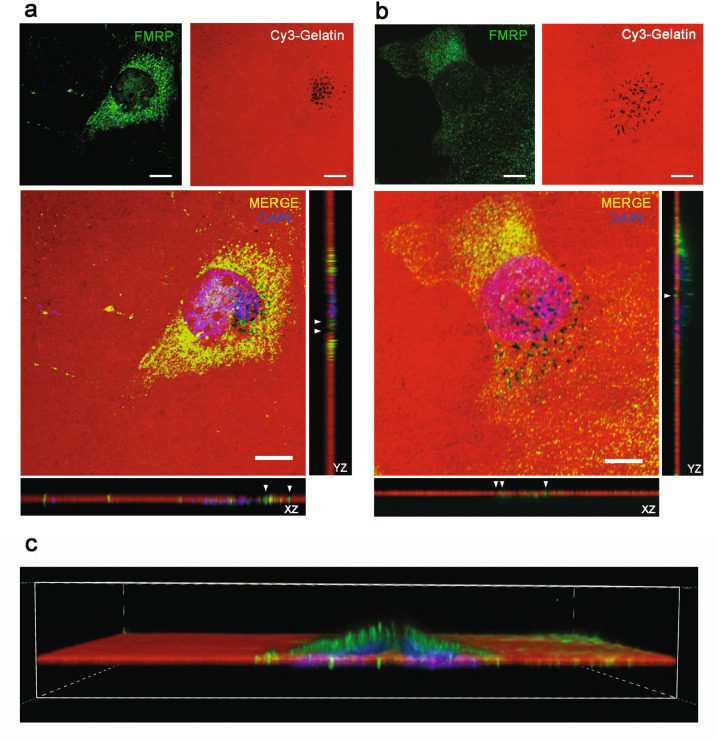
FMRP is localized inside invadopodia of HuH28 and HuCCT cells. **a** A total of 40% confluent HuH28 cells were sown in Cy3-gelatin coated chambers slides and incubated for 19 h for a GDA. Then an IF was performed, and the images were captured using a confocal microscope. Cy3-gelatin is red, FMRP staining green and DAPI staining blue. Representative images of a 60× magnification field (field 15 of 27 total fields). Scale bars: 10 μm. To the right and under the MERGE image, orthogonal views along Y (YZ) and X (XZ) axes are shown, respectively. White arrowheads indicate FMRP staining inside black areas of Cy3-gelatin (degradation areas). **b** 40% confluent HuCCT cells starved for 24 h in 2% FBS medium were sown in Cy3-gelatin coated chambers slides, treated for 16 h with the MMP inhibitor GM6001 and then incubated for 2 h without MMP inhibition for a GDA. IF and image acquisitions were performed as reported for HuH28. Representative images of a 60× magnification field (field 18 of 28 total fields). Scale bars: 10 μm. To the right and under the MERGE image, orthogonal views along Y (YZ) and X (XZ) axes are shown respectively. White arrowheads indicate FMRP staining inside black areas of Cy3-gelatin (degradation areas). **c** 3D reconstruction of Z stack (along *Y* axis) reported in panel (**b**).

Proteolytic degradation of extracellular matrix (ECM) is a critical step during cancer cell invasion and invadopodia are the protrusions situated in the ventral cell surface and often under the nucleus, that mediate cell adhesion to, degradation of, and invasion into ECM in tumor cells [30]. To verify the presence of FMRP also in these protrusions, we performed a Cy3-gelatin degradation assay (GDA) and 19 h later we detected FMRP protein by confocal imaging both in HuH28 (Fig. 4a) and HuCCT cells (Fig. 4b). Since HuCCT cells were found to be poorly prone to gelatin degradation (GD) (24 h of incubation showed no or limited GD, data not shown), we stimulated invadopodia formation with starvation and MMP deprivation before GDA (see “Material and Methods”). In both HuH28 (Fig. 4a) and HuCCT cells (Fig. 4b) FMRP was found to localize in mature-late stages of invadopodia [31] that are inside the black area of gelatin (GD), and in mature-early stages or precursor-late stages of invadopodia [31] that reside in the non-degraded gelatin. The orthogonal view (Z stack) of FMRP staining showed that FMRP foci are localized in the ventral part of the cells, mainly under the nucleus (Fig. 4, white arrowheads), consistent with the invadopodia localization [30]. In particular, in HuCCT cells, which are thicker than HuH28 cells, 3D confocal reconstruction clearly showed FMRP in invadopodia protrusions in the ventral and sub-nuclear area of the cell (Fig. 4c).

Interestingly, FMRP and F-actin double staining showed that FMRP foci frequently localize in degradation spots with no F-actin puncta and only rarely in F-actin enriched mature-late invadopodia (Supplementary Fig. 2, white arrowheads in orthogonal view). Of note, the localization of FMRP in invadopodia seems to be common also to other cancer cells, such as the A375 melanoma cells (Supplementary Fig. 3), for which the role of FMRP in cancer progression has been previously investigated [16].

### FMRP silencing affects migration, invasion, and invadopodia formation in iCCA cells

**Fig. 5:**
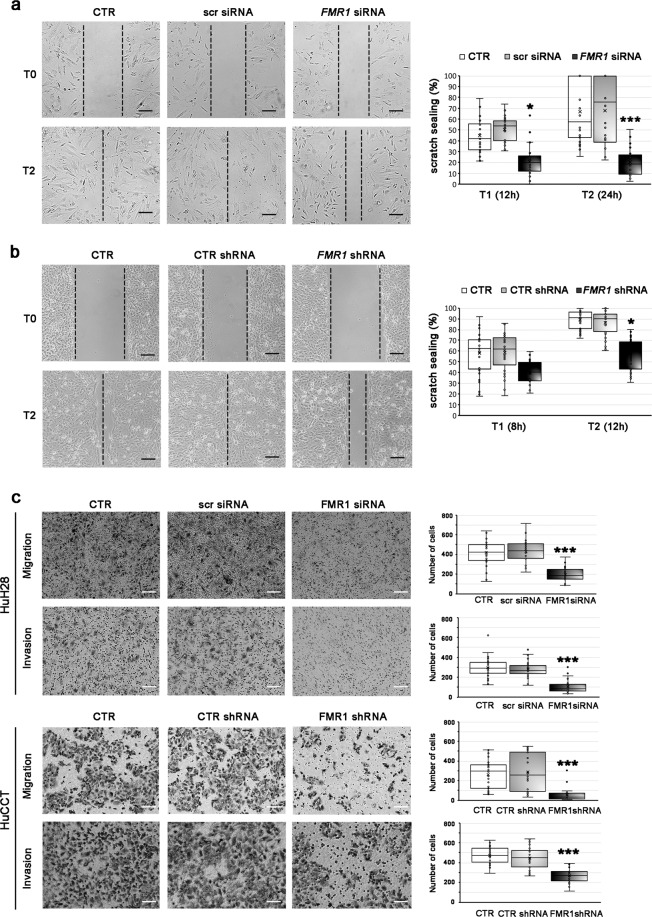
Silencing of FMRP affects migration and invasion in HuH28 and HuCCT cells. **a** Confluent cell monolayers of HuH28 cells untransfected (CTR) or transfected with *FMR1* siRNAs (*FMR1* siRNA) or scrambled siRNA (scr siRNA) were wounded and after 0 (T0), 12 (T1), or 24 (T2) h the scratch spreads were evaluated. In the left panels, one defined area per group at initial (T0) and final (T2) times is shown. Scale bars: 300 μm. In 10 defined areas per group and in three independent experiments, the scratch sealing was quantified and shown (in %) in the boxplot (right panel). The boxes represent quartiles; the ×, the median; the dots, the individual data points and the whiskers, the variability beyond the upper and lower quartiles. *p < 0.05; ***p < 0.001 compared to CTR or scr siRNA (Student’s *t* test). **b** Confluent cell monolayers of HuCCT cells untransfected (CTR) or stable transfected with *FMR1* shRNAs (*FMR1* shRNA) or control shRNA (CTR shRNA) were wounded and after 0 (T0), 8 (T1), or 12 (T2) h the scratch spreads were evaluated. In the left panels, one defined area per group at initial (T0) and final (T2) times is shown. Scale bars: 300 μm. In 10 defined areas per group and in three independent experiments, the scratch sealing was quantified and shown (in %) in the boxplot (right panel). The boxes represent quartiles; the ×, the median; the dots, the individual data points and the whiskers, the variability beyond the upper and lower quartiles. *p < 0.05 compared to CTR or CTR shRNA (Student’s *t* test). **c** Previously cited HuH28 or HuCCT cells were sown into trans-well motility chambers (50,000 cells/well; upper panels) or Matrigel-coated trans-well invasion chambers (100,000 cells/well; lower panels) and after 24 h migrating or invading cells were counted. In left panels, a representative field (4× magnification) per group is shown. Scale bars: 300 μm. In right panels, cells present in ten magnification fields per group and per experiment were counted. Data derive from three independent experiments. The boxes represent quartiles; the ×, the median; the dots, the individual data points and the whiskers, the variability beyond the upper and lower quartiles. ***p < 0.001 compared to CTR or scr siRNA or CTR shRNA (Student’s *t* test).

To verify the role of FMRP in motility and invasion in iCCA cells, FMRP expression was knocked down both in HuH28 and HuCCT cells. Because HuH28 cells have a low duplication time, it was not possible to perform a stable knock down, therefore HuH28 cells were transiently transfected with specific *FMR1* siRNAs and FMRP knockdown was confirmed by western blotting (Supplementary Fig. 4a). On the contrary, we succeeded in generating HuCCT cells with stable expression of *FMR1* specific shRNAs using a lentiviral vector and found a consistent downregulation of FMRP and *FMR1* mRNA levels (Supplementary Fig. 4b). FMRP knockdown in HuCCT cells resulted in a certain attenuation of growth as subcutaneous xenografts in nude mice, but this effect did not reach statistical significance (Supplementary Fig. 4c). However, reduction of FMRP expression did have a marked effect on cell migration and invasion in vitro. Indeed, FMRP knockdown reduced cell motility into a wounded area of confluent cultures in HuH28 (Fig. 5a) and HuCCT cells (Fig. 5b). Accordingly, in trans-well migration assays, motility of HuH28 and HuCCT was significantly lower in *FMR1*-knockdown cells compared to untransfected controls, or in scrambled siRNA or CTR shRNA transfected cells (Fig. 5c, migration panels). Similarly, in trans-well invasion assays, *FMR1*-knockdown HuH28 or HuCCT cells were consistently and significantly less invasive than the respective controls (Fig. 5c, invasion panels). Conversely, the overexpression of FMRP in HuCCT cells (Supplementary Fig. 5a, b) increases both migration and invasion capabilities, in trans-well assays (Supplementary Fig. 5c). Notably, the survival rate of both iCCA cell lines in FMRP silenced versus control conditions did not change, excluding a relevant effect of FMRP on proliferation and/or cell death during migration and invasion assays (Supplementary Fig. 6).

Altogether, these data confirm the involvement of FMRP in modulating the migratory and invasive properties of iCCA cells.

**Fig. 6:**
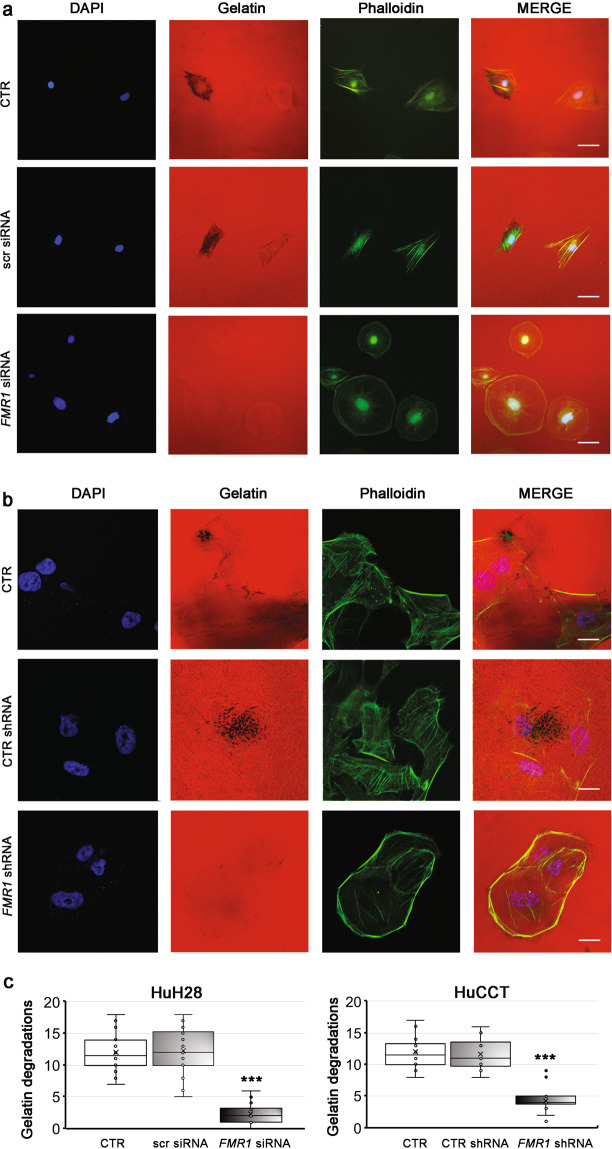
Silencing of FMRP reduces the ability of HuH28 and HuCCT cells to degrade the Cy3-gelatin in GDA assays. **a** HuH28 cells were untransfected (CTR) or transfected with three specific *FMR1* siRNAs (*FMR1* siRNA) or with a scrambled nonspecific siRNA (scr siRNA), incubated for 72 h and then sown on Cy3-gelatin coated chamber slides (4000 cells for chamber) for a GDA assay. After 24 h, cells were fixed and stained with phalloidin (F-actin) and DAPI (nuclei). Representative images of 20× magnification fields of nuclei (blue), Cy3-gelatin (red), and F-actin (green). In the last right panels, merged fluorescence was shown. Scale bars: 60 μm. **b** HuCCT cells were untransfected (CTR) or stable transfected with a specific *FMR1* shRNA (*FMR1* shRNA) or with a control shRNA (CTR shRNA) and then starved for 24 h in 2% FBS medium. The cells were then sown on Cy3-gelatin coated chamber slides (4000 cells for chamber), treated for 16 h with MMP inhibitor GM6001 and then incubated without MMP inhibitor for a GDA assay. After 4 h, cells were fixed and stained with phalloidin (F-actin) and DAPI (nuclei). Representative images of 60× magnification fields of nuclei (blue), Cy3-gelatin (red), and F-actin (green). In the last right panels, merged fluorescence was shown. Scale bars: 20 μm. **c** Quantification of GDs (dark areas in Cy3-gelatin) present in 10 fields of both HuH28 (left panel) and HuCCT (right panel) cell lines. *N* = 3. The boxes represent quartiles; the ×, the median; the dots, the individual data points and the whiskers, the variability beyond the upper and lower quartiles. ***p < 0.001 compared to CTR or scr siRNA or CTR shRNA (Student’s *t* test).

To investigate the involvement of FMRP in invadopodia formation we performed GDAs in FMRP silenced HuH28 and HuCCT cells. FMRP silencing significantly reduced (>60%) the cellular capability to degrade Cy3-gelatin, both in HuH28 (Fig. 6a, c, left panel) and HuCCT cells (Fig. 6b, c, right panel). Of note, in FMRP silenced HuH28 cells the less invasive behavior is accompanied by a particular change in the cell morphology, with cells appearing to have a rounder shape compared to the more front-rear and migrating morphology of control cells (Supplementary Fig. 7). However, in HuCCT cells the changes in the cellular shape were not observed (data not shown), probably due to stimulation conditions pre-GDA assays.

### FMRP binds mRNAs encoding key proteins involved in invadopodia formation and function

**Fig. 7:**
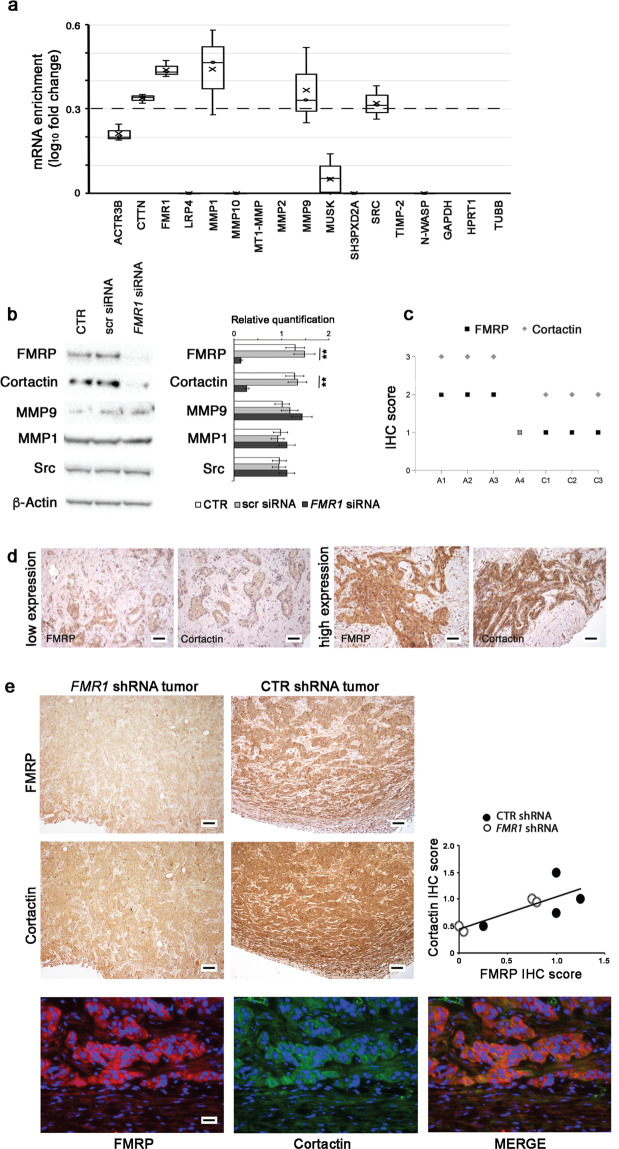
FMRP binds cortactin mRNA and regulates its expression. **a** Cytoplasmic extracts of HuH28 cells were used for immunoprecipitation experiments using the specific anti-FMRP antibody ab17722 (IP FMRP) or nonspecific IgG antibodies (IP IgG). The co-immunoprecipitated RNAs were extracted from each IP and *ACTR3B, CTTN, FMR1, LRP4, MMP1, MMP10, MT1-MMP, MMP2, MMP9, MUSK, SH3PXD2A, SRC, TIMP-2, N-WASP, GAPDH, HPRT1,* and *TUBB* mRNAs were quantified by NanoString nCounter^®^ approach. The enrichment of these mRNAs (expressed as log_10_ fold change) in FMRP IP versus IgG IP is reported in the boxplot, where only the positive values are shown. *N* = 3. Dashed line represents the confidence threshold chosen for log_10_ fold change that correspond to a fold change = 2. **b** HuH28 cells were untransfected (CTR) or transfected with three specific *FMR1* siRNAs (*FMR1* siRNA) or with a scrambled nonspecific siRNA (scr siRNA) and incubated for 72 h. Representative images of a western blot probed with anti-FMRP, anti-Cortactin, anti-MMP9, anti-MMP1, anti Src, and anti-β-Actin antibodies from protein extracts of the three cellular conditions (left panel). In right panel, a quantitation of FMRP, Cortactin, MMP9, MMP1, and Src expressions, compared to β-Actin expression, is shown. Columns, mean; bars, SE. **p < 0.01 compared to CTR or scr siRNA (Student’s *t* test). **c** IHC data were obtained by The Human Protein Atlas library. Four iCCA samples (A1–A4) and three healthy livers (C1–C3) were evaluated for both Cortactin and FMRP expression and scored from +0 to +3. **d** Cortactin expression was evaluated by IHC in three human iCCAs with low FMRP expression and three human iCCAs with high FMRP expression. Representative images of FMRP and Cortactin immunostaining in a FMRP low expression sample (left panels) and a FMRP high expression sample (right panels) were shown. Scale bars: 150 μm. **e** Representative images of FMRP and Cortactin IHCs in xenograft tumors from *FMR1* shRNA (left panels) or CTR shRNA (right panels) transfected HuCCT cells. Original magnification 10×; scale bars: 250 μm. FMRP and Cortactin IHC expression in *FMR1* shRNA and CTR shRNA xenograft tumors are highly correlated (right panel); E, *ρ* = 0.34, *p < 0.05 with Spearman’s correlation Test. Double-IF labeling experiments show most cells co-expressing FMRP and Cortactin proteins (lower panels). FMRP was stained in red, Cortactin in green and nuclei in DAPI (blue). Original magnification 40×; scale bars: 60 μm.

To explore the possibility that FMRP could represent a regulator of local mRNA metabolism in pseudopodia/invadopodia, we immunoprecipitated FMRP complex from HuH28 cells with a specific commercially available anti-FMRP polyclonal antibody (ab17722) and, in FMRP co-immunoprecipitated RNA pool, we checked the presence of mRNAs encoding for key proteins involved in invadopodia formation and function (Fig. 7a) using the NanoString nCounter^®^ technology (Supplementary Fig. 8a).

As reported in Fig. 7a, *CTTN*, *FMR1*, *MMP1*, *MMP9*, and *SRC* mRNAs are consistently (fold change > 2.0 and log_10_fold change > 0.3; dashed threshold line in Fig. 7a) and significantly enriched in FMRP co-IP mRNA pool compared to IgG-IP nonspecific mRNAs, demonstrating that these mRNAs are targets of FMRP in HuH28 cells (Fig. 7a). As negative control, the three housekeeping genes *GAPDH*, *HPRT1*, and *TUBB* were not co-immunoprecipitated with FMRP complex (Fig. 7a). To verify if FMRP is able to modulate the expression of the proteins encoded by the FMRP mRNA targets, we analysed protein extracts from control HuH28 cells (untransfected CTR or scr siRNA transfected cells) or FMRP silenced cells (*FMR1* siRNA) performing a Western blot. Interestingly, FMRP knockdown significantly reduced the expression of Cortactin, a marker of mature invadopodia, while had not effect on the abundance of the other proteins involved in invadopodia formation (Fig. 7b). This decrease was clearly visible in FMRP silenced HuH28 cells also by IF experiments (Supplementary Fig. 8b).

In agreement with these findings, FMRP and Cortactin expressions showed a direct correlation in iCCA samples of TGCA data set (Fig. 7c), and in the cohort of iCCA samples analysed in this study (Fig. 7d). Finally, this direct correlation between FMRP and Cortactin expression was also observed in xenograft tumors from nude mice injected with stably transfected *FMR1* shRNA or CTR shRNA HuCCT cells. In these tumors, FMRP knockdown was associated with reduced Cortactin staining, and regions of intense FMRP immunostaining corresponded to high levels of Cortactin signal (Fig. 7e).

## Discussion

Although some clinicopathological parameters, including lymph node metastasis and tumor differentiation, have been shown to be prognostic factors for iCCA [32, 33], the identification of molecular prognostic markers and the understanding of the pathogenic mechanisms involved in iCCA are still far off [34, 35].

Our work revealed that FMRP could have a pathogenetic role in iCCA progression. Indeed, we showed that FMRP is highly expressed in human iCCA tissues, and its expression levels significantly correlate with the metastatic nature of iCCA tissues. Accordingly, the modulation of FMRP levels in iCCA cell lines by silencing or overexpression impairs or increases their invasive and migratory capacities, which play a central role in metastatization [36, 37].

About the molecular mechanism by which FMRP could have such a pathogenetic effect, we observed that it is particularly enriched in the cells localized at the tumor-host interface (named “invasive tumor front” ITF) [38], with a peculiar intracellular dotted and under-membrane localization which resembles the neuronal pattern [39] and which suggests a common function for FMRP in all polarized cells: the local regulation of mRNA metabolism (localization, stability, and local protein synthesis) at the leading edge.

mRNA localization and local protein synthesis have a fundamental importance for the establishment and maintenance of cancer cell polarity during migration and invasion [40–42]. These processes are initiated by cellular protrusions named lamellipodia/filopodia and invadopodia, that have been increasingly recognized as important drivers of local invasion in metastasis [43, 44]. In polarized invading cells, these highly localized events are regulated by specific factors (mRNAs and RNA binding proteins), which often exist at high concentrations at the leading edges of cells [45–48]. Some of these RNA binding proteins have been well characterized as key players in cancer progression and metastatization, such as IGF2BPs [49, 50] and IMPs [51].

Our data demonstrate that FMRP might represent another of these important RNA binding proteins. Indeed, by fluorescent immunocytochemistry, immunogold TEM, and time-lapse live imaging we showed that FMRP localizes, in form of large granules, inside pseudopodia protrusions generating at the leading edge of migrating cells. The FMRP granules were well characterized in neurons and correspond to large ribonucleoprotein (RNP) complexes containing translationally inhibited mRNAs [39], that move along dendrites through the motor proteins kinesin KIF3C [52, 53], dynein [53] and myosin Va [54, 55]. Similar granules, containing FMRP and translationally silent mRNAs, were also observed in pseudopodial protrusions of migrating fibroblasts [56].

The crucial role of some RNA binding proteins (such as IMP proteins) in regulating local protein synthesis in invadopodia has been previously demonstrated [51]. Here we demonstrated for the first time that also FMRP localizes inside invadopodia, both in iCCA and melanoma cells.

**Fig. 8:**
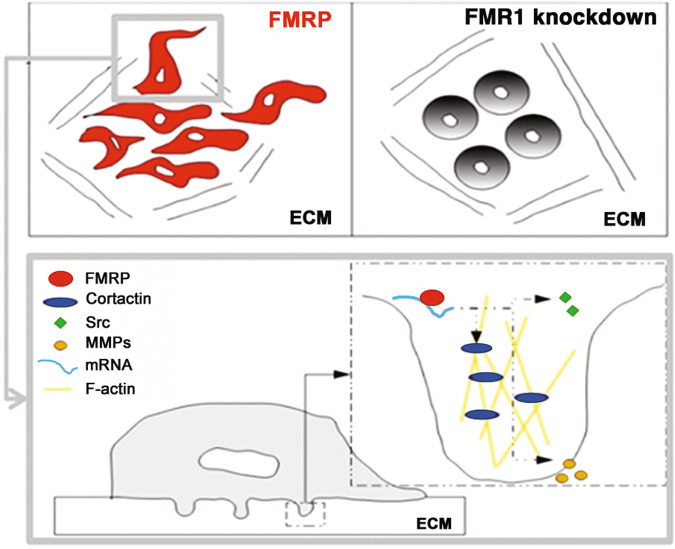
Working model for FMRP action in invadopodia. FMRP in invading iCCA cells could regulate local metabolism (mRNA stability, transport, translation etc.) of specific mRNAs (such as *Cortactin* or *MMP* mRNAs) in invadopodia, favoring invadopodia formation and membrane plasticity at the leading edges of cancer cells during invasion processes (left panel with enlarged box). Silencing of FMRP reduced cell invasiveness, reducing their front-rear morphology and their ability to migrate and to degrade the ECM (right panel).

In our GDA experiments, the partial correspondence between FMRP staining and F-actin puncta, as well as the partial correspondence between FMRP puncta and gelatin degradation areas, suggests an early and/or late role of FMRP in invadopodia formation, during those preparatory stages, in which local protein synthesis and mRNA metabolism regulation could be of key importance to guarantee the correct assembly and function of these protrusions. This hypothesis was confirmed by the observation that FMRP is able to bind several mRNAs codifying key proteins for invadopodia formation and function, such as Cortactin, Metalloproteinase 1 and 9 and Src, in addition to binding its own mRNA as observed also in neurons [57, 58]. Despite *MMP9* and *CTTN* are known mRNA targets of FMRP in neurons [11, 59–61], the mRNAs identified in our study represent the first FMRP mRNA targets in iCCA cells. Due to the importance of their encoded proteins in orchestrating the molecular events that occurs in invadopodia during their cycle (formation, stabilization, MMP secretion, and dissolution), these mRNAs represent interesting effector molecules through which FMRP may have an impact on invadopodia formation and membrane plasticity at the leading edges of invading iCCA cells (Fig. 8).

FMRP can regulate different aspects of mRNA metabolism of its target mRNAs, such as their transport [52, 62], stability [63–66], editing [67, 68] and translation [10, 69, 70]. Our evidence that FMRP knockdown decreases Cortactin expression but does not affect the expression of the other bound mRNAs, suggests that FMRP could exert its action as translational or stability regulator for Cortactin mRNA, but could regulate aspects of mRNA metabolism that don’t affect protein expression (e.g., editing or transport) for the other targeted mRNAs. In agreement with this idea, we observed a direct correlation between FMRP and Cortactin expression in iCCA tissues, iCCA cell lines and in xenograft tumors from *FMR1* silenced HuCCT cells injected in nude mice.

In conclusion, despite further studies are required to demonstrate the role of FMRP as molecular prognostic marker of iCCA, our data shaded light on a possible FMRP-dependent pathogenetic mechanism in iCCA. Specifically, cancer cells could take opportunistic advantage from FMRP overexpression, increasing their migratory and invasive capabilities through the local regulation of key proteins involved in plasma membrane plasticity and invadopodia formation and function at the leading edges.

## Materials and methods

### Human tissues collection and patient information

iCCA and eCCA samples from patients used in this study were provided by Pathology Department of Campus Bio-Medico University (Rome, Italy). All experiments involving human specimens are conformed to the principles described in the NMA declaration of Helsinki. All human tissues were collected following standardized procedures and informed consent was obtained for all specimens linked with clinical data, according to procedures approved by the Institutional Ethical Board of the Campus Bio-Medico University. The histopathological diagnoses of the tumors were described according to the World Health Organization (WHO) International Classification of Disease for Oncology. Only patients with another neoplasm or with insufficient material for analysis were excluded. The medical records of all patients were examined to obtain clinical and histopathological information.

### Immunohistochemical analysis

FMRP IHC was performed using polyclonal antibody ab17722 (Abcam, Cambridge, MA) (1:50 dilution), while Cortactin IHC was performed using monoclonal anti-Cortactin 771716 (R&D System, Minneapolis, MN, USA) (1:200 dilution), according to the protocol reported in Supplementary Information.

### Cell cultures

The SV40-transformed (i.e., immortalized) intrahepatic normal human biliary epithelial cell line H69, the human iCCA cell lines HuH28 and HuCCT and the eCCA cell lines KMBC, TFK1, and MzChA1 have been described before [71] and were generously shared by Dr. J Banales (BioDonostia Institute and CIBERehd, San Sebastian, Spain). Cells were cultured in Dulbecco’s modified Eagle’s medium F12 (DMEM-F12, Thermo Fisher Scientific, Waltham, MA, USA) supplemented with 10% fetal bovine serum (FBS) (Gibco, MD, USA) and antibiotics (50 U/ml penicillin, 50 μg/ml streptomycin, Thermo Fisher Scientific, Waltham, MA, USA) at 37 °C in a humidified atmosphere of 5% CO2 (Heraeus, Hanau, Germany). Before use, each cell culture was tested for mycoplasma contamination.

### RT-qPCR

Total RNA from cell lines was extracted using the automated Maxwell system from Promega (Madison, WI). Quantitative reverse transcription PCR was performed as reported, and gene expression was normalized relative to that of the housekeeping gene *histone H3* as described [72]. Primer sequences for qPCR were: for *FMR1* sense 5ʹ-ACAGAGGAAGAGAGGGAGAGC-3ʹ and antisense 5ʹ-CCTTTCATTATTGCAGTCAACTC-3ʹ; for *H3*: sense 5ʹ-AAAGCCGCTCGCAAGAGTGCG-3ʹ and antisense 5ʹ-CCTTTCATTATTGCAGTCAACTC-3ʹ.

### Western blotting

Cellular lysates were prepared as previously reported [16] and western blotting was performed according to the protocol reported in Supplementary Information.

### Immuno transmission electron microscopy

Immuno Transmission Electron Microscopy (iTEM) was performed as reported [16] using anti-FMRP (1:5) and anti-Cortactin (1:10) antibodies. The grids were then incubated for 1 h with goat anti-rabbit IgG (1:5) conjugated with 25 nm colloidal-gold particles (British Biocell, Cardiff, United Kingdom) and with anti-mouse IgG (1:5) conjugated with 15 nm colloidal-gold particles (British Biocell, Cardiff, United Kingdom), counterstained in uranyl acetate to underline the cell morphology, and observed with EM 109 electron microscope (Zeiss, Oberkochen, Germany).

### Transient silencing of FMRP in HuH28 cells

Small interfering RNA-mediated silencing of FMRP were performed with *FMR1*-specific siRNAs from Life Technologies (AM 16708, ID #10824, #10919 and #11010). As a nonspecific control, a scramble siRNA was used (#4390843; Life Technologies, Waltham, MA). siRNAs were transfected into HuH28 cells using Lipofectamine RNAiMAX (Life Technologies, Waltham, MA), according to manufacturer’s instructions. Transfections were carried out in six-well plates at 40% confluence with 90 pmol of siRNAs, and HuH28 cells were harvested after 24, 48, or 72 h.

### Stable silencing of FMRP in HuCCT cells

Lentiviral vectors (pGFP-shLenti) with *FMR1*-specific or control CTR-shRNAs (cat. # TL312955) were from OriGene Technologies (Rockville, MD). Lentiviral particles were prepared in 293 T cells using the Lenti-Vpak packaging kit (cat. # TR30037), also from OriGene Technologies, following the manufacturer’s instructions. HuCCT cells were sown in six wells plates (25 × 10^4^ cells/well) and were incubated with *FMR1*-shRNA, or CTR-shRNAs lentiviral particles for 36 h. Then cells were washed, and 36 h later puromycin (0.25 μg/ml) (Sigma, St. Louis, MO) was added to the medium to start selection.

### Tumor xenograft model

The subcutaneous tumor model was established in male athymic nude mice (8 weeks old, *n* = 6 per group) (Envigo, Valencia, Spain) essentially as described [73]. Briefly, cells (1 × 10^7^) were resuspended in 50 μl phosphate buffer (PBS) plus 50 μl Matrigel (from Corning, Corning, NY) and subcutaneously injected in the right flank of mice. Tumor growth was measured with a Vernier caliper using the formula V = (length x width2)/2. Animal studies were approved by the Animal Care Committee of the University of Navarra (protocol number 159/14). No randomization method was applied, because no different experimental groups with different treatments were processed.

### Transient overexpression of FMRP in HuCCT cells

The pRRL-h*FMR1*-GFP lentiviral vector expressing the Iso7 isoform of human FMRP fused to eGFP was a generous gift by Prof. Suzanne Zukin (Albert Einstein College of Medicine, NY, USA) to CB. The pRRL-h*FMR1*-GFP or the empty eGFP vector as control were transiently transfected into HuCCT cells using Lipofectamine 3000 (Life Technologies), according to manufacturer’s instructions. Transfections were carried out in six-well plates at 70% confluence with 3 μg of pRRL-h*FMR1*-GFP vector (or eGFP vector) and HuCCT cells were harvested after 24, 48, or 72 h.

### Time-lapse live imaging

HuCCT cells overexpressing FMRP-GFP or eGFP were sown in fibronectin-coated chambers and placed in epifluorescence microscope incubator for a time-lapse live imaging experiment as described in Supplementary Information.

### Scratch assay

Scratch assay was performed as previously described [16] and detailed in Supplementary Information.

### Trans-well migration and invasion assays

Trans-well migration and invasion assays were performed as described [16] and detailed in Supplementary Information.

### Immunofluorescence (IF)

Immunofluorescence experiments were performed as reported in Supplementary Information.

### GDA

Eight well chamber slides were coated with 0.2% Cy3-gelatin (ECM671, Merck KGaA, Darmstadt, Germany) according to the datasheet. 40% confluent HuH28 cells were detached, plated in completed media (4000 cells for well) and left to degrade the Cy3-gelatin for 19 or 24 h, as described [74]. For GDA experiments with HuCCT, before seeding, 40% confluent cells were starved for 24 h in 2% FBS D-MEM medium. Then the cells were detached and sown on Cy3-gelatin coated chamber slides (4000 cells for chamber) in completed media containing 25 μM MMP inhibitor (GM6001, Merck KGaA, Darmstadt, Germany) to block GD. After 16 h, MMP inhibitor was removed and the cells were left to degrade Cy3-gelatin for 2 or 4 h in completed media. Then IF experiments were performed in the above described conditions. Images were captured with a NIKON TiA1 confocal as a z-stack series using the NIS element software and processed with Fiji software (US National Institutes of Health).

### Immunoprecipitation

HuH28 Cells were lysed as reported [13] and IP was performed using Dynabeads Protein G immunoprecipitation kit (Life Technologies, Waltham, MA) according to manufacturer’s protocol. RNA was Trizol (Life technologies, Waltham, MA) extracted, isopropanol precipitated and quantified by BioSpectrometer^®^ (Eppendorf).

### NanoString nCounter^®^ analysis

One hundred ng of total RNAs from co-IP RNAs or inputs (HuH28 cytoplasmic extracts) were subjected to NanoString nCounter^®^ analysis, using a custom gene set containing 14 selected target genes *ACTR3B*, *CTTN*, *FMR1*, *LRP4*, *MMP1*, *MMP10*, *MT1-MMP*, *MMP2*, *MMP9*, *MUSK*, *SH3PXD2A*, *SRC*, *TIMP-2*, *N-WASP*, and three housekeeping genes, *GAPDH*, *HPRT1*, and *TUBB*. NanoString nCounter^®^ analysis was performed following the manufacturer’s protocol (www.nanostring.com). Raw counts for each mRNA were normalised and analysed using nSolver Analysis Software (nSAS) to obtained differential expression analysis of 17 mRNAs in FMRP co-IP RNA samples versus IgG co-IP RNA samples.

### Statistical analysis

Results are presented as mean ± SE or mean ± SD or as boxplots showing the individual data points, the median values and the variability beyond the upper and lower quartiles, with experiment numbers indicated in the figure legends. Statistical significance was assessed using the Mann–Witney test or the Student’s *t* test, according to non-normal or normal distribution respectively. *P* < 0.05 was accepted as statistically significant. The investigators were blinded to the group allocation during the experiments and/or when assessing the outcome.

## Supplementary information

Supplementary Information

Movie 1 (FITC)

Movie 1 (FITC+PC)

Movie 2 (FITC)

Movie 2 (FITC+PC)

Movie 3 (FITC)

Movie 3 (FITC+PC)

## References

[1] Clements O, Eliahoo J, Kim JU, Taylor-Robinson SD, Khan SA (2020). Risk factors for intrahepatic and extrahepatic cholangiocarcinoma: a systematic review and meta-analysis. J Hepatol.

[2] Sirica AE, Gores GJ, Groopman JD, Selaru FM, Strazzabosco M, Wei Wang X (2019). Intrahepatic cholangiocarcinoma: continuing challenges and translational advances. Hepatology.

[3] Rizvi S, Kan SA, Hallemeier CL, Kelley RK, Gores GJ (2018). Cholangiocarcinoma—evolving concepts and therapeutic strategies. Nat Rev Clin Oncol.

[4] Bagni C, Tassone F, Neri G, Hagerman R (2012). Fragile X syndrome: causes, diagnosis, mechanisms, and therapeutics. J Clin Invest.

[5] Bagni C, Zukin RS (2019). A synaptic perspective of fragile X syndrome and autism spectrum disorders. Neuron.

[6] Bagni C, Oostra BA (2013). Fragile X syndrome: from protein function to therapy. Am J Med Genet A.

[7] Bhakar AL, Dolen G, Bear MF (2012). The pathophysiology of fragile X (and what it teaches us about synapses). Annu Rev Neurosci.

[8] Gross C, Barry-Kravis EM, Bassell GJ (2012). Therapeutic strategies in fragile X syndrome: dysregulated mGluR signaling and beyond. Neuropsychoparmacology.

[9] Miyashiro KY, Beckel-Mitchener A, Purk TP, Becker KG, Barret T, Liu L (2003). RNA cargoes associating with FMRP reveal deficits in cellular functioning in Fmr1 null mice. Neuron.

[10] Darnell JC, Van Driesche SJ, Zhang C, Hung KY, Mele A, Fraser CE (2011). FMRP stalls ribosomal translocation on mRNAs linked to synaptic function and autism. Cell.

[11] Ascano M, Mukherjee N, Bandaru P, Miller JB, Nusbaum JD, Corcoran DL (2012). FMRP targets distinct mRNA sequence elements to regulate protein expression. Nature.

[12] Maurin T, Lebrigand K, Castagnola S, Paquet A, Jarjat M, Popa A (2018). HITS-CLIP in various brain areas reveals new targets and new modalities of RNA binding by fragile X mental retardation protein. Nucleic Acids Res.

[13] Lucà R, Averna M, Zalfa F, Vecchi M, Bianchi F, La Fata G (2013). The fragile X protein binds mRNAs involved in cancer progression and modulates metastasis formation. EMBO Mol Med.

[14] Xing Z, Zeng M, Hu H, Zhang H, Hao Z, Long Y (2016). Fragile X mental retardation protein promotes astrocytoma proliferation via the MEK/ERK signaling pathway. Oncotarget.

[15] Srivastava A (2015). A novel link between FMR gene and the JNK pathway provides clues to possible role in malignant pleural mesothelioma. FEBS Open Bio.

[16] Zalfa F, Panasiti V, Carotti S, Zingariello M, Perrone G, Sancillo L (2017). The fragile X mental retardation protein regulates tumor invasiveness-related pathways in melanoma cells. Cell Death Dis.

[17] Di Grazia A, Marafini I, Pedini G, Di Fusco D, Laudisi F, Dinallo V (2020). The fragile X mental retardation protein regulates RIP1K and colorectal cancer resistance to necroptosis. Cell Mol Gastroenterol Hepatol.

[18] Schultz-Pedersen S, Hasle H, Olsen JH, Friedrich U (2001). Evidence of decreased risk of cancer in individuals with fragile X. Am J Med Genet.

[19] Kalkunte R, Macarthur D, Morton R (2007). Glioblastoma in a boy with fragile X: an unusual case of neuroprotection. Arch Dis Child.

[20] Kurokawa Y, Matoba R, Takemasa I, Nakamori S, Tsujie M, Nagano H (2003). Molecular features of non-B, non-C hepatocellular carcinoma: a PCR-array gene expression profiling study. J Hepatol.

[21] Liu Y, Zhu X, Zhu J, Liao S, Tang Q, Liu K (2007). Identification of differential expression of genes in hepatocellular carcinoma by suppression subtractive hybridization combined cDNA microarray. Oncol Rep.

[22] Li Y, Tang Y, Ye L, Liu B, Liu K, Chen J (2003). Establishment of a hepatocellular carcinoma cell line with unique metastatic characteristics through in vivo selection and screening for metastasis-related genes through cDNA microarray. J Cancer Res Clin Oncol.

[23] Li L, Zeng Q, Bhutkar A, Galván JA, Karamitopoulou E, Noordermeer D (2018). GKAP acts as a genetic modulator of NMDAR signaling to govern invasive tumor growth. Cancer Cell.

[24] Dianat N, Dubois-Pot-Schneider H, Steichen C, Desterke C, Leclerc P, Raveux A (2014). Generation of functional cholangiocyte-like cells from human pluripotent stem cells and HepaRG cells. Hepathology.

[25] Kusaka Y, Tokiwa T, Sato J (1988). Establishment and characterization of a cell line from a human cholangiocellular carcinoma. Res Exp Med.

[26] Weed SA, Parsons JT (2001). Cortactin: coupling membrane dynamics to cortical actin assembly. Oncogene.

[27] Arjonen A, Kaukonen R, Ivaska J (2011). Filopodia and adhesion in cancer cell motility. Cell Adhes Migr.

[28] Macgrath SM, Koleske AJ (2012). Cortactin in cell migration and cancer at a glance. J Cell Sci.

[29] Chen WT, Wang JY (1999). Specialized surface protrusions of invasive cells, invadopodia and lamellipodia, have differential MT1-MMP, MMP-2, and TIMP-2 localization. Ann N. Y Acad Sci.

[30] Murphy DA, Courtneidge SA (2011). The ‘ins’ and ‘outs’ of podosomes and invadopodia: characteristics, formation and function. Nat Rev Mol Cell Biol.

[31] Beaty BT, Condeelis J (2014). Digging a little deeper: the stages of invadopodium formation and maturation. Eur J Cell Biol.

[32] Wang Y, Li J, Xia Y, Gong R, Wang K, Yan Z (2013). Prognostic nomogram for intrahepatic cholangiocarcinoma after partial hepatectomy. J Clin Oncol.

[33] Mavros MN, Economopoulos KP, Alexiou VG, Pawlik TM (2014). Treatment and prognosis for patients with intrahepatic cholangiocarcinoma: systematic review and meta-analysis. JAMA Surg.

[34] Banales JM, Cardinale V, Carpino G, Marzioni M, Andersen JB, Invernizzi P (2016). Cholangiocarcinoma: current knowledge and future perspectives consensus statement from the European Network for the Study of Cholangiocarcinoma (ENS-CCA). Nat Rev Gastroenterol Hepatol.

[35] Rizvi S, Gores GJ (2017). Emerging molecular therapeutic targets for cholangiocarcinoma. J Hepatol.

[36] Hanahan D, Weinberg RA (2011). Hallmarks of cancer: the next generation. Cell.

[37] Yilmaz M, Christofori G (2009). EMT, the cytoskeleton, and cancer cell invasion. Cancer Metastasis Rev.

[38] Christofori G (2006). New signals from the invasive front. Nature.

[39] Ferrari F, Mercaldo V, Piccoli G, Sala C, Cannata S, Achsel T (2007). The fragile X mental retardation protein-RNP granules show an mGluR-dependent localization in the post-synaptic spines. Mol Cell Neurosci.

[40] Hsieh AC, Liu Y, Edlind MP, Ingolia NT, Janes MR, Sher A (2012). The translational landscape of mTOR signalling steers cancer initiation and metastasis. Nature.

[41] Silvera D, Formenti SC, Schneider RJ (2010). Translational control in cancer. Nat Rev Cancer.

[42] Stumpf CR, Ruggero D (2011). The cancerous translation apparatus. Curr Opin Genet Dev.

[43] Bravo-Cordero JJ, Hodgson L, Condeelis J (2012). Directed cell invasion and migration during metastasis. Curr Opin Cell Biol.

[44] Eddy RJ, Weidmann MD, Sharma VP, Condeelis JS (2017). Tumor cell invadopodia: invasive protrusions that orchestrate metastasis. Trends cell Biol.

[45] Insall RH, Machesky LM (2009). Actin dynamics at the leading edge: from simple machinery to complex networks. Dev Cell.

[46] Ridley AJ (2011). Life at the leading edge. Cell.

[47] Pollard TD, Cooper JA (2009). Actin a central player in cell shape and movement. Science.

[48] Liao G, Mingle L, Van De Water L, Liu G (2015). Control of cell migration through mRNA localization and local translation. Wiley Interdiscip Rev RNA.

[49] Taniuchi K, Furihata M, Hanazaki K, Saito M, Saibara T (2014). IGF2BP3-mediated translation in cell protrusions promotes cell invasiveness and metastasis of pancreatic cancer. Oncotarget.

[50] Bell JL, Wachter K, Mühleck B, Pazaitis N, Köhn M, Lederer M (2013). Insulin-like growth factor 2 mRNA-binding proteins (IGF2BPs): post-transcriptional drivers of cancer progression?. Cell Mol Life Sci.

[51] Vikesaa J, Hansen TV, Jønson L, Borup R, Wewer UM, Christiansen J (2006). RNA-binding IMPs promote cell adhesion and invadopodia formation. EMBO J.

[52] Davidovic L, Jaglin XH, Lepagnol-Bestel AM, Tremblay S, Simonneau M, Bardoni B (2007). The fragile X mental retardation protein is a molecular adaptor between the neurospecific KIF3C kinesin and dendritic RNA granules. Hum Mol Genet.

[53] Dictenberg JB, Swanger SA, Antar LN, Singer RH, Bassell GJ (2008). A direct role for FMRP in activity-dependent dendritic mRNA transport links filopodial-spine morphogenesis to fragile X syndrome. Dev Cell.

[54] Lindsay AJ, McCaffrey MW (2014). Myosin Va is required for the transport of fragile X mental retardation protein (FMRP) granules. Biol Cell.

[55] Ohashi S, Koike K, Omori A, Ichinose S, Ohara S, Kobayashi S (2002). Identification of mRNA/protein (mRNP) complexes containing Puralpha, mStaufen, fragile X protein, and myosin Va and their association with rough endoplasmic reticulum equipped with a kinesin motor. J Biol Chem.

[56] Mili S, Moissoglu K, Macara IG (2008). Genome-wide screen reveals APC-associated RNAs enriched in cell protrusions. Nature.

[57] Schaeffer C, Bardoni B, Mandel JL, Ehresmann B, Ehresmann C, Moine H (2001). The fragile X mental retardation protein binds specifically to its mRNA via a purine quartet motif. EMBO J.

[58] Didiot MC, Tian Z, Schaeffer C, Subramanian M, Mandel JL, Moine H (2008). The G-quartet containing FMRP binding site in *FMR1* mRNA is a potent exonic splicing enhancer. Nucleic Acids Res.

[59] Janusz A, Milek J, Perycz M, Pacini L, Bagni C, Kaczmarek L (2013). The Fragile X mental retardation protein regulates matrix metalloproteinase 9 mRNA at synapses. J Neurosci.

[60] Sidhu H, Dansie LE, Hickmott PW, Ethell DW, Ethell IM (2014). Genetic removal of matrix metalloproteinase 9 rescues the symptoms of fragile X syndrome in a mouse model. J Neurosci.

[61] Gkogkas CG, Khoutorsky A, Cao R, Jafarnejad SM, Prager-Khoutorsky M, Giannakas N (2014). Pharmacogenetic inhibition of eIF4E-dependent Mmp9 mRNA translation reverses fragile X syndrome-like phenotypes. Cell Rep.

[62] Kao DI, Aldridge GM, Weiler IJ, Greenough WT (2010). Altered mRNA transport, docking, and protein translation in neurons lacking fragile X mental retardation protein. Proc Natl Acad Sci U.S.A.

[63] D’Hulst C, De Geest N, Reeve SP, Van Dam D, De Deyn PP, Hassan BA (2006). Decreased expression of the GABAA receptor in fragile X syndrome. Brain Res.

[64] Zalfa F, Eleuteri B, Dickson KS, Mercaldo V, De Rubeis S, Di (2007). A new function for the fragile X mental retardation protein in regulation of PSD-95 mRNA stability. Nat Neurosci.

[65] Zhang M, Wang Q, Huang Y (2007). Fragile X mental retardation protein FMRP and the RNA export factor NXF2 associate with and destabilize Nxf1 mRNA in neuronal cells. Proc Natl Acad Sci U.S.A.

[66] De Rubeis S, Bagni C (2010). Fragile X mental retardation protein control of neuronal mRNA metabolism: insights into mRNA stability. Mol Cell Neurosci.

[67] Bhogal B, Jepson JE, Savva YA, Pepper AS, Reenan RA, Jongens TA (2011). Modulation of dADAR-dependent RNA editing by the Drosophila fragile X mental retardation protein. Nat Neurosci.

[68] Filippini A, Bonini D, Lacoux C, Pacini L, Zingariello M, Sancillo L (2017). Absence of the fragile X mental retardation protein results in defects of RNA editing of neuronal mRNAs in mouse. RNA Biol.

[69] Zalfa F, Giorgi M, Primerano B, Moro A, Di Penta A, Reis S (2003). The fragile X syndrome protein FMRP associates with BC1 RNA and regulates the translation of specific mRNAs at synapses. Cell.

[70] Napoli I, Mercaldo V, Boyl PP, Eleuteri B, Zalfa F, De Rubeis S (2008). The fragile X syndrome protein represses activity-dependent translation through CYFIP1, a new 4E-BP. Cell.

[71] Zach S, Birgin E, Ruckert F (2015). Primary cholangiocellular carcinoma cell lines. J Stem Cell Res Transpl.

[72] Elizalde M, Urtasun R, Azkona M, Latasa MU, Goñi S, García-Irigoyen O (2014). Splicing regulator SLU7 is essential for maintaining liver homeostasis. J Clin Invest.

[73] Bárcena-Varela M, Caruso S, Llerena S, Álvarez-Sola G, Uriarte I, Latasa MU (2019). Dual targeting of histone methyltransferase G9a and DNA-methyltransferase 1 for the treatment of experimental hepatocellular carcinoma. Hepatology.

[74] Vira VA, Kenneth MY, Susette C, Mueller ECM (2009). Degradation assays for analyzing local cell invasion. Methods in molecular biology. Extracell Matrix Protoc.

