# Supplementary material for: Fragile X mental retardation protein in intrahepatic cholangiocarcinoma: regulating the cancer cell behavior plasticity at the leading edge
Source: Oncogene. 2021 May 20;40(23):4033–49. doi: 10.1038/s41388-021-01824-3 (PMC8195741; doi:10.1038/s41388-021-01824-3)
Supplement: Supplementary file 1 — Supplementary Information [file 41388_2021_1824_MOESM1_ESM.pdf]

SUPPLEMENTARY MATERIALS AND METHODS

Western blotting.

Membranes were incubated using specific antibodies for FMRP (1:500, #ab17722, Abcam, Cambridge, MA), β-Actin (1:1000, #ab8227, Abcam, Cambridge, MA), Cortactin (1:1000, #771716, R&D System, Inc Minneapolis, MN), MT1-MMP (1:1000, #5H2, R&D System, Inc Minneapolis, MN), MMP9 (1:1000, #4A3, Novus Biologicals, Littleton, CO), MMP1 (1:1000, #36665 R&D System, Inc Minneapolis, MN), Src (1:10 000, #36D10, Cell Signaling Technology, Leiden, WZ), and signal was detected using an enhanced chemiluminescence kit (GE Healthcare, Buckinghamshire, UK) and acquired by ChemiDoc™ MP System (Bio-Rad, California, USA).

Immunohistochemical analysis.

Immunohistochemical detection was performed with labelled polymer in accordance with the standard UltraVision AP detection system (Thermo Fisher Scientific). Immunohistochemical reactions were visualized by DAB as the chromogen from MACH 1 Universal HRP-Polymer Detection (Biocare Medical, Concord, MA, USA). Hematoxylin was used as counterstains in order to visualize nuclei. A semi-quantitative approach was used to evaluate FMRP and Cortactin protein expression. The immunostaining was evaluated by using an open source plugin for Image J[1]. For each sample four fields were analysed (magnification 20×). Since CCA might have wide desmoplastic reaction, we performed a manual selection of neoplastic tissue on previously acquired fields in order to exclude the cancer stroma from automated analysis. The selected areas from each field were scored as 0, 1, 2 by ImageJ. For correlative purpose, a median score was derived for each sample.

Immunofluorescence (IF).

HuH28 or HuCCT cells were cultured on fibronectin-coated chamber slides at low concentration (40% confluence), washed with PBS and fixed with 4% paraformaldehyde (in PBS) for 15 min. Cells

were permeabilized and blocked with 0,25% Triton X-100 and 2% normal serum (in PBS) for 20 min, and then incubated in 1% normal serum, 0,1% Triton X-100 (in PBS) with the appropriate primary antibodies for 2 h at room temperature. The primary antibodies were used at the following concentrations: 1:50 anti-FMRP, 1:50 anti-MT1-MMP and 1:20 anti-Cortactin. The cells were washed in PBS and then incubated with an anti-rabbit Alexa Fluor® 568 or an anti-mouse Alexa Fluor® 488 conjugated secondary antibodies (ThermoFisher Scientific, Waltham, MA, USA) diluted 1:200 in PBS for 1 h at 37°C. Fluorescently double-labeled cells were mounted in ProLong® Diamond Antifade Mounting with DAPI (ThermoFisher Scientific, Waltham, MA, USA). Images were taken by a FV1000 confocal microscope (Olympus, Tokyo, Japan) or a TiA1 confocal microscope (NIKON, Amsterdam, Netherlands) and processed with Fiji software (US National Institutes of Health).

Time-lapse live imaging.

FITC signals corresponding to FMRP-GFP fusion protein or eGFP protein (green) and phase-contrast signals (PC, grey) were acquired in 20× magnification fields every 10 minutes for 12 h and the 72 total acquisitions were assembled to produce live imaging movies with a speed of 4 frames per second.

Scratch assay.

A monolayer of HuH28 or HuCCT (control or FMRP silenced) cells was scratched using a pipette tip through the central axis of the plate. Migration of the cells into the scratch was digitally documented 0, 12 and 24 h (for HuH28 cells) or 0, 8 and 12 h (for HuCCT cells) after being made, and relative migratory activity was calculated based on the cell-free areas.

Trans-well migration and invasion assays.

For migration assay, 5×10^4 (or 2.5×10^4) HuH28 or HuCCT cells (control, FMRP silenced or FMRP

overexpressed), previously overnight starved in D-MEM F12 + 2% FBS, were added to the top chambers of 24-transwell plates (Corning Costar, Kennebunk, ME, USA; 8 μm pore size) and D-MEM F12 media + 20% FBS was added to the bottom chambers. After 24 h, cells were fixed for 20 min with 4% paraformaldehyde and stained for 40 min with 0.1% crystal violet (Sigma, St. Louis, MO, USA). Top (non-migrated) cells were removed with a cotton swab and bottom (migrated) cells were counted. Invasion assays were performed as for migration assays, using 1 $\times 10^5$ cells (or 5 $\times 10^4$) and matrigel-coated transwells (Corning Costar). Experiments were repeated a minimum of three times.

Cell block

CTR shRNA or *FMR1* shRNA stable transfected HuCCT cells from confluent T-25 flasks were pelleted at 1200 rpm for 5 minutes and the cellular pellets were formalin fixed and paraffin embedded according to standard protocols. 3μm sections from these cell blocks were used for IHC experiments.

MTT assay

Control or FMRP-silenced HuH28 or HuCCT cells were seeded on 96-well plates (5 000 cells/well) and a MTT assay (#30006, Biotium, Fremont, USA) was performed according to the manufacturer protocol. After 0, 4, 8, 12, 16, 20, 24 h, cell viability was evaluated by formazan absorbance measurement with a TECAN spectrophotometer (AG, Switzerland). Normalized absorbance values were obtained subtracting signal absorbance (570 nm) to background absorbance (630 nm).

SUPPLEMENTARY REFERENCES

1. Varghese F, Bukhari AB, Malhotra R, De A. IHC Profiler: An Open Source Plugin for the Quantitative Evaluation and Automated Scoring of Immunohistochemistry Images of Human Tissue Samples. Plos one 2014; 9: e96801.

SUPPLEMENATRY FIGURES

Supplementary Figure 1

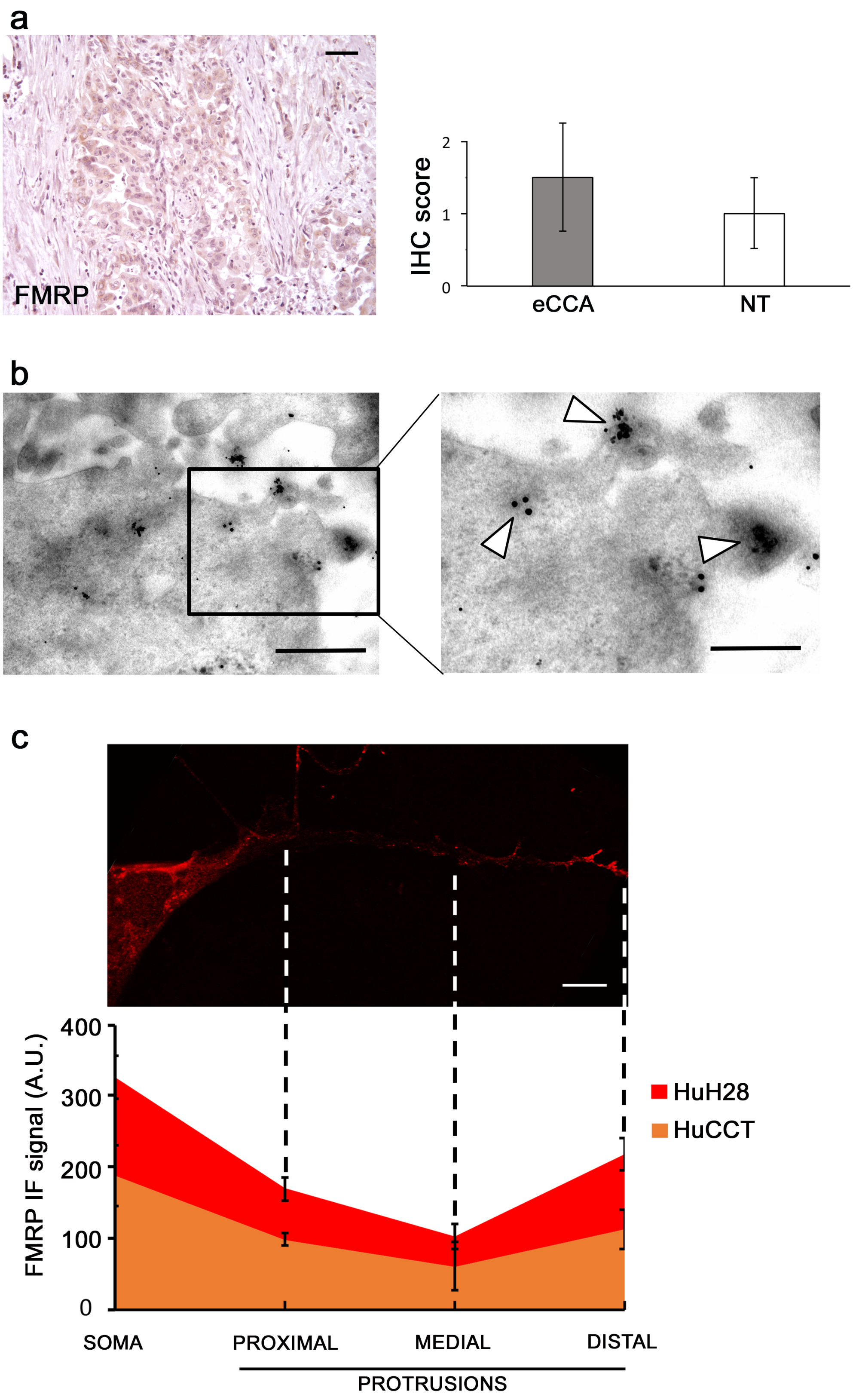

Supplementary Figure 2

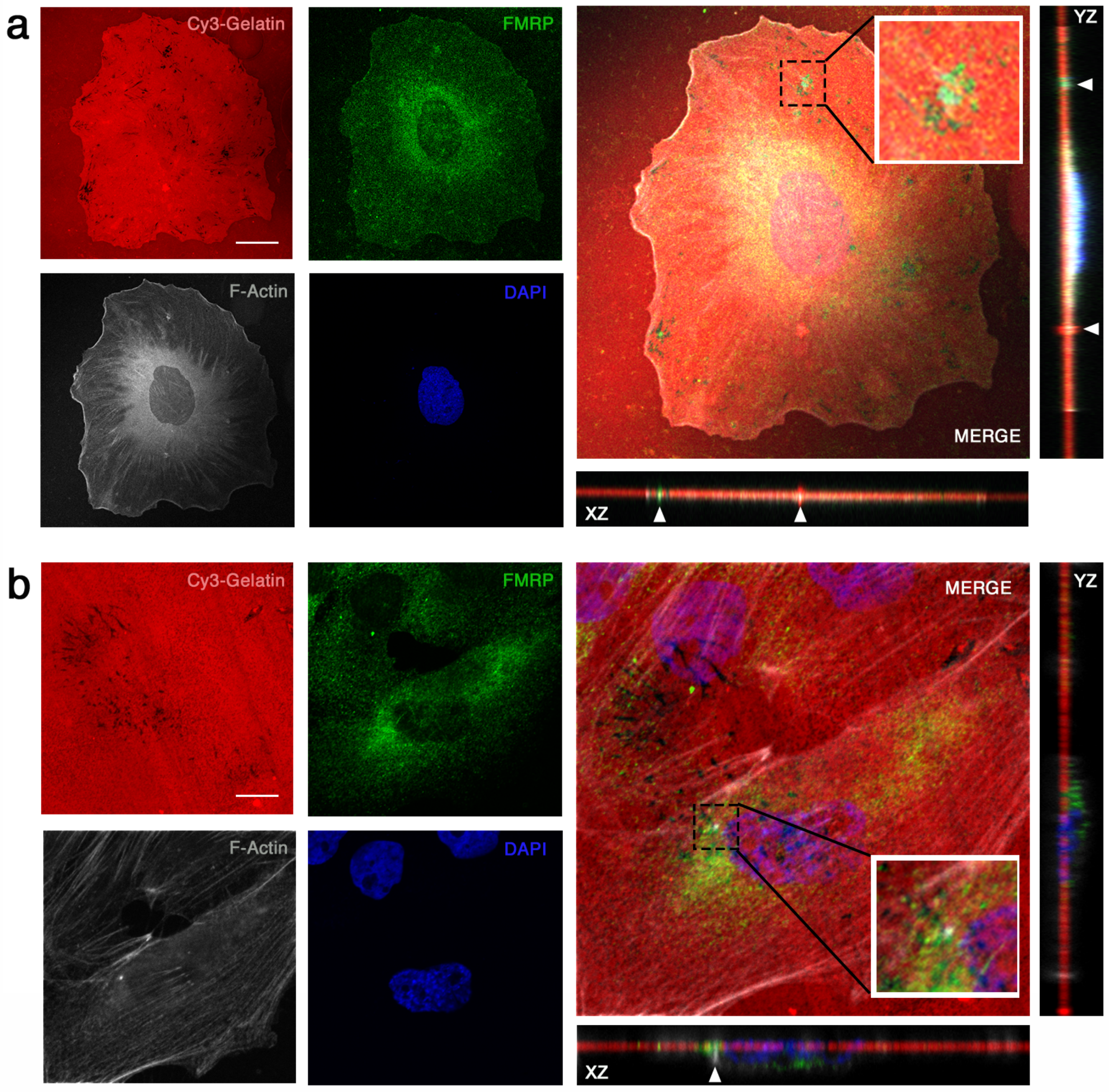

Supplementary Figure 3

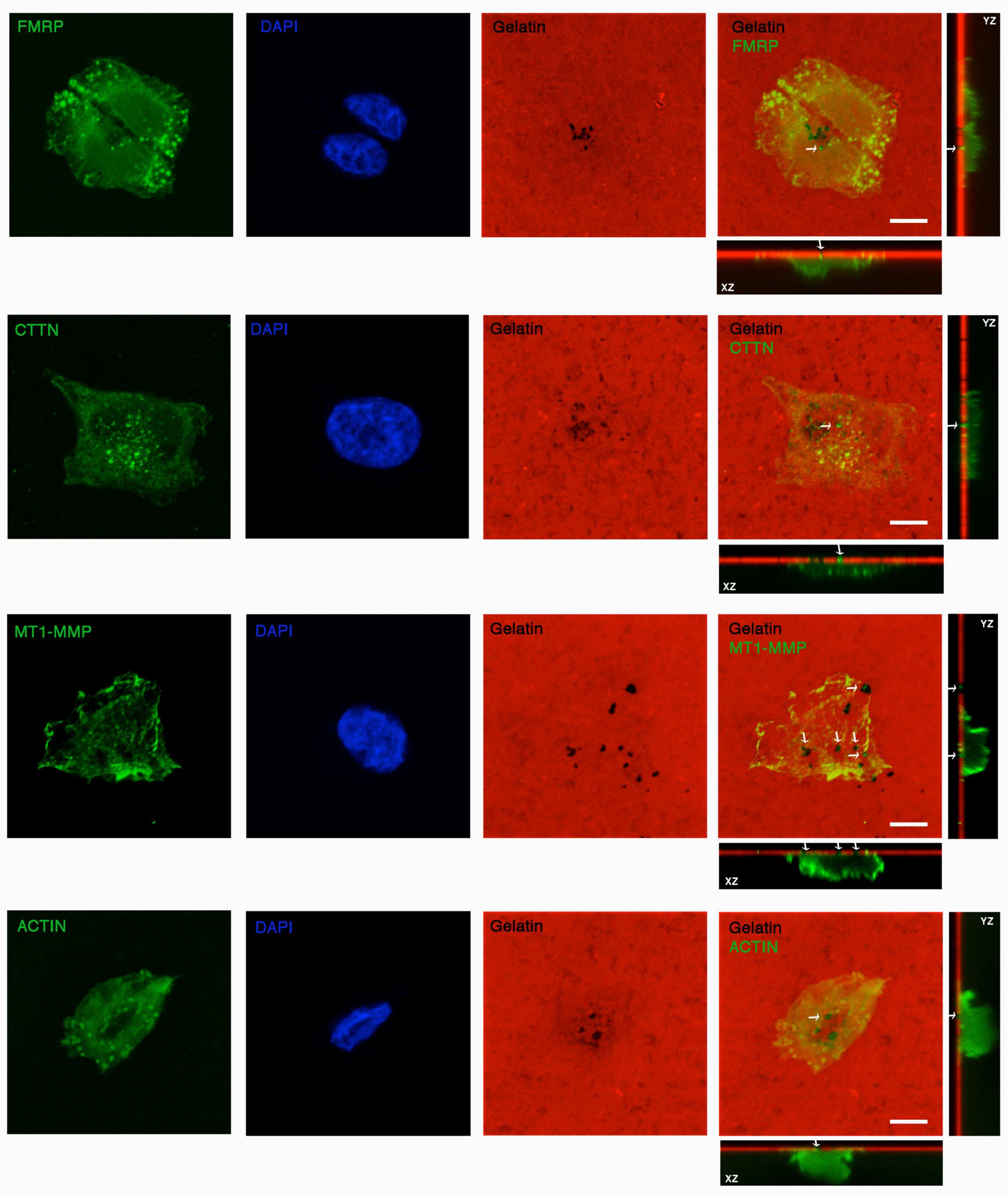

Supplementary Figure 4

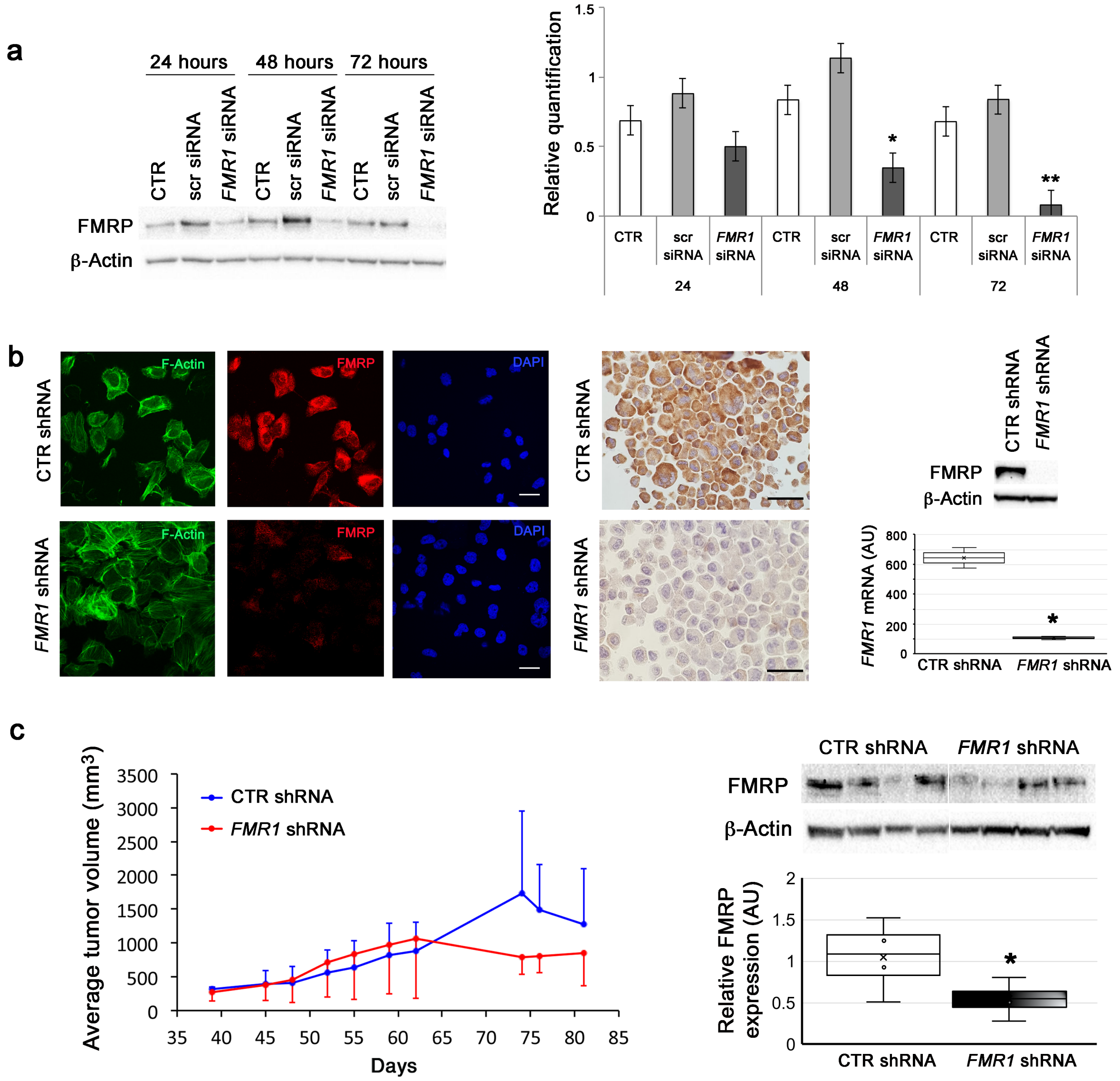

Supplementary Figure 5

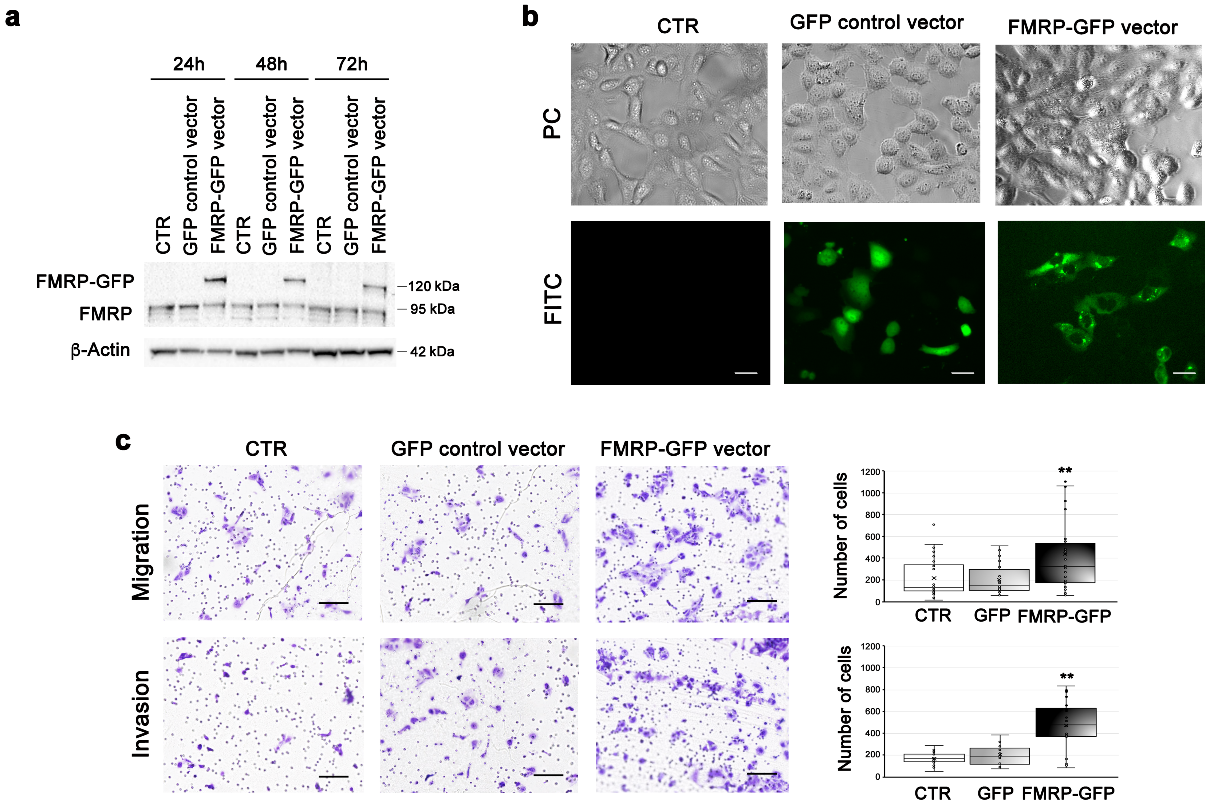

Supplementary Figure 6

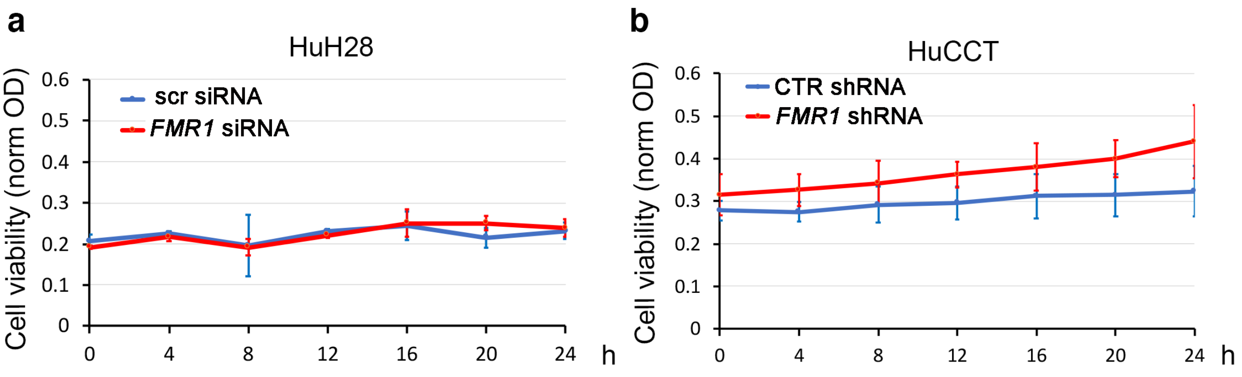

Supplementary Figure 7

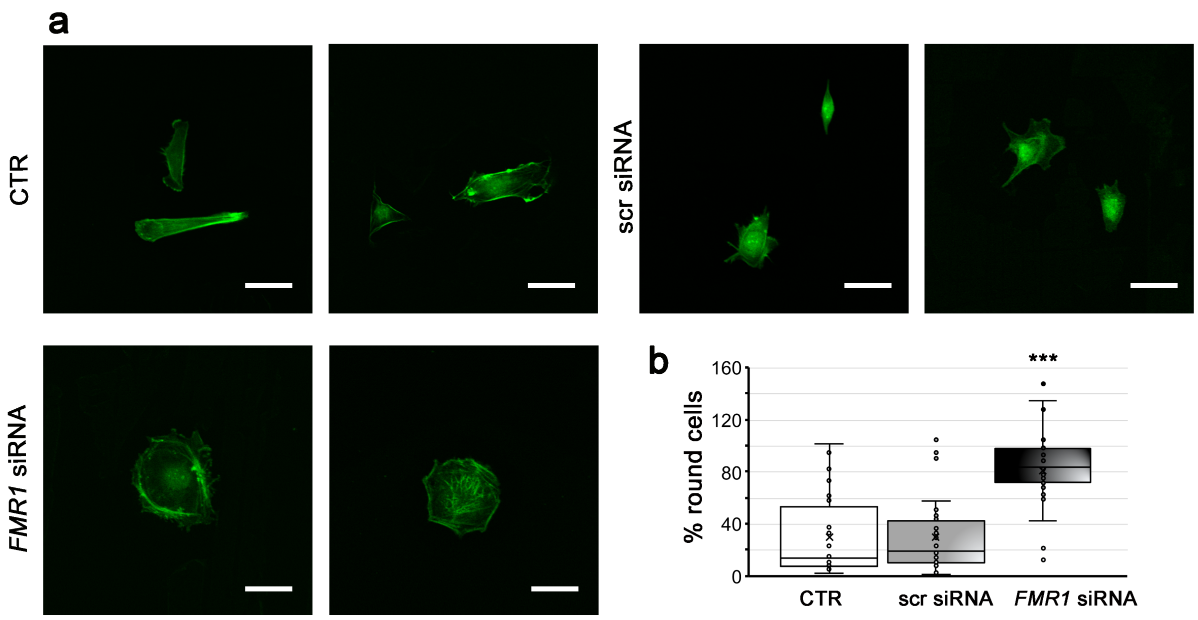

Supplementary Figure 8

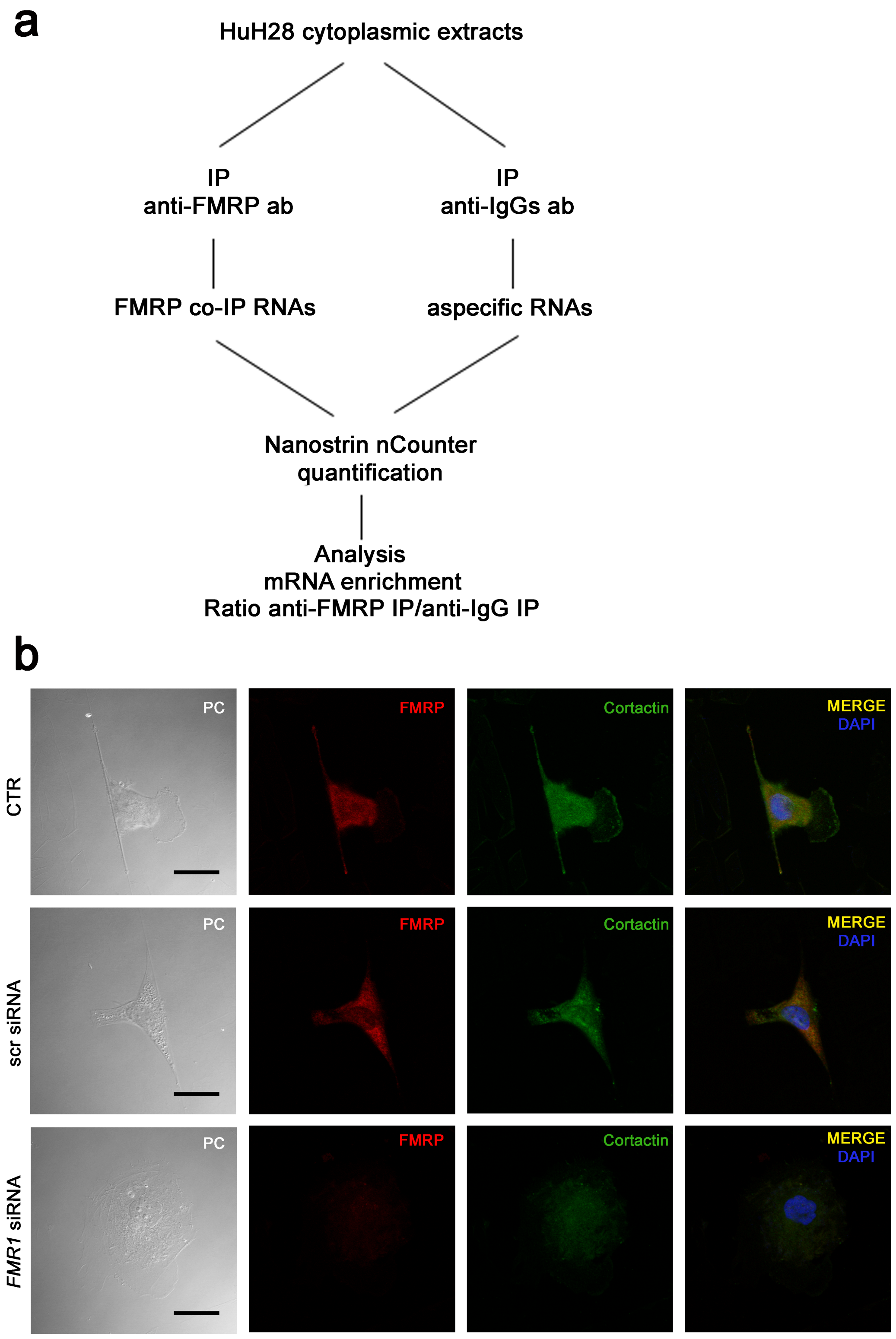

SUPPLEMENTARY FIGURE LEGENDS

Supplementary Figure 1. FMRP immunostaining in eCCA tissues and FMRP immunogold in HuH28 cells.

a) FMRP expression was evaluated by IHC in 17 human eCCA samples and 7 non-tumoral hepatic tissues (NT). In left panel, a representative image of FMRP IHC in an eCCA sample was reported. Calibration bars: 50 μm. Each sample was scored by using a semi-automated method and the IHC score of eCCA and NT tissues was reported in the right histogram. The columns represent median values and the bars, standard deviations. **b)** Ultrathin sections of HuH28 cellular pellets were subjected to immunogold EM with specific FMRP and Cortactin antibodies. The box in left panel indicates the high power field in right panel. 25 nm gold particles (FMRP) and 15 nm gold particles (Cortactin) co-localized at the base of membrane protrusions (white arrowheads) at the leading edge of HuH28 cells. Original magnification × 23 000. Scale bars: 1μm (left panel); 500 nm (right panel). **c)** In 10 different IF confocal acquisitions of HuH28 or HuCCT cells (one of which is shown in upper panel), red signal (corresponding to FMRP staining) was quantified in different parts of cells (soma, proximal protrusions, medial protrusions or distal protrusions) and the average values were graphed in the lower panel with their standard deviations. Original magnification 40×. Scale bar: 10μm.

Supplementary Figure 2. Partial FMRP/F-actin colocalization in invadopodia of HuH28 and HuCCT cells.

a) 40% confluent HuH28 cells were sown in Cy3-gelatin coated chambers slides and incubated for 19 h for a GDA. Then an IF was performed, and the images were captured using a confocal microscope. Cy3-gelatin is red, FMRP staining green, F-actin staining grey and DAPI staining blue. A field (field 17 of 30 total fields) is shown as representative image (original magnification 60×).

Scale bar: 50 µm. To the right and under the MERGE image, orthogonal views along Y (YZ) and X (XZ) axes are shown respectively. White arrowheads indicate FMRP and F-actin colocalizations inside black areas of Cy3-gelatin (degradation areas). In the enlarged box, a magnification of one of these colocalization areas is shown. **b)** 40% confluent HuCCT starved for 24 h in 2% FBS medium were sown in Cy3-gelatin coated chambers slides, stimulated for 16 h with the MMP inhibitor GM6001 and then incubated without MMP inhibition for 2 h for a GDA. IF and image acquisitions were performed as reported for HuH28. A field (field 12 of 39 total fields) is shown as representative image (original magnification 60×). Scale bar: 20 µm. To the right and under the MERGE image, orthogonal views along Y (YZ) and X (XZ) axes are shown respectively. White arrowheads indicate FMRP and F-actin colocalizations inside black areas of Cy3-gelatin (degradation areas). In the enlarged box, a magnification of one of these colocalization areas is shown.

Supplementary Figure 3. FMRP, Cortactin, MT1-MMP and F-actin localization in invadopodia of A375 melanoma cells.

40% confluent A375 cells were sown in Cy3-gelatin coated chambers slides and incubated for 19 h for a GDA. Then an IF was performed, and the images were captured using a confocal microscope. Cy3-gelatin is red, FMRP, Cortactin (CTTN), MT1-MMP and F-actin staining green, and DAPI staining blue. Original magnification 60×. Scale bars: 10 µm. To the right and under the MERGE image, orthogonal views along Y (YZ) and X (XZ) axes are shown respectively. White arrows indicate FMRP, Cortactin (CTTN), MT1-MMP and F-actin localizations inside black areas of Cy3-gelatin (degradation areas).

Supplementary Figure 4. FMRP silencing in HuH28 and HuCCT cell lines.

a) HuH28 cells were untransfected (CTR) or transfected with three specific *FMR1* siRNAs (*FMR1* siRNA) or with a scrambled aspecific siRNA (scr siRNA) and incubated for 24, 48 or 72 h. Western blots probed with anti-FMRP antibody or with anti-β-Actin show the expression of the two proteins

in the three different conditions (left panel). A quantitation of FMRP expression, with respect β-Actin expression, is shown in right panel. Columns, mean; bars, SE. *$p < 0.05$ and **$p < 0.01$ compared to CTR or scr siRNA (Student's t-test). **b)** HuCCT cells were stable transfected with *FMR1* specific shRNAs (*FMR1* shRNA) or with a control shRNA (CTR shRNA) and IF (left panels) or IHC (central panels) was performed. In left panels representative images of 40× magnification fields for each cellular condition. FMRP is red, F-actin green and DAPI blue. Scale bars: 50 μm. In central panels, representative images of IHC FMRP staining on HuCCT cell block sections. Original magnification: 40×. Scale bars: 70 μm. In right top panel, western blot probed with anti-FMRP antibody or with anti-β-Actin shows the expression of the two proteins in protein extracts from CTR shRNA or *FMR1* shRNA stable transfected cells. A quantitation of *FMR1* mRNA expression in CTR shRNA or *FMR1* shRNA stable transfected cells, performed with Nanostring technology, is shown as boxplot in right bottom panel. The boxes represent quartiles, the ×, the median and the whiskers, the variability beyond the upper and lower quartiles. *, $p < 0.05$ compared to CTR shRNA (Student's t-test). **c)** Athymic nude mice were subcutaneously injected with CTR shRNA or *FMR1* shRNA stable transfected HuCCT cells and the xenograft tumor volume was monitored for 80 days. In left panel, the plot of average tumor volume for the two types of injected mice is shown (n=4). Bars represent SD. In right panel, a representative western blotting for FMRP and β-Actin, using protein extracts (15 μg) from the eight xenograft tumors monitored in left panel. In the boxplot, the quantification of FMRP with respect β-Actin signals in the two types of xenograft tumors (CTR shRNA and *FMR1* shRNA) is shown. The boxes represent quartiles; the ×, the median; the dots, the individual data points and the whiskers, the variability beyond the upper and lower quartiles. *, $p < 0.05$ compared to CTR shRNA (Student's t-test).

Supplementary Figure 5. Migration and invasion assays on FMRP overexpressed HuCCT cells. **a)** HuCCT cells were untransfected (CTR) or transfected with a eGFP vector (GFP control vector) or

transfected with a vector expressing an FMRP isoform (Iso7) fuses to eGFP (FMRP-GFP vector) and incubated for 24, 48 or 72 h. Western blot probed with anti-FMRP antibody or with anti-β-Actin shows the expression of three proteins (FMRP, FMRP-GFP fusion protein and β-Actin) in the three different conditions. **b)** Previously cited HuCCT cells were sown in fibronectin-coated chambers and after 24 h, phase contrast (PC, upper panels) and FITC signal (lower panels) in live cells were acquired with an epifluorescence microscope. Representative images for each cellular condition are shown. GFP or FMRP-GFP are green and PC is grey. Original magnification 40×. Scale bars: 30 µm **c)** Previously cited HuCCT cells were sown into trans-well motility chambers (25 000 cells/well; upper panel) or Matrigel-coated trans-well invasion chambers (50 000 cells/well; lower panel) and after 24 h, migrating or invading cells were counted. In left panels, a representative field (10× magnification) per group is shown. Scale bars: 150 µm. In right panels, cells present in ten magnification fields per group and per experiment were quantify as boxplot. The boxes represent quartiles; the ×, the median; the dots, the individual data points and the whiskers, the variability beyond the upper and lower quartiles. **$p < 0.01$, compared to CTR or GFP control vector (Student's t-test). Data derive from three independent experiments.

Supplementary Figure 6. MTT assay on HuH28 and HuCCT cells during migration/invasion time frames.

Control or *FMR1*-silenced HuH28 **(a)** or HuCCT **(b)** cells were sown in 96-well plates (5 000 cell/wells for HuH28 or 8 000 cell/wells for HuCCT) and after 4, 8, 12, 16, 20 or 24 h cell viability was measured with a MTT assay. N = 3.

Supplementary Figure 7. FMRP silencing affects cell morphology in migrating HuH28 cells.

a) HuH28 cells were untransfected (CTR) or transfected with three specific *FMR1* siRNAs (*FMR1* siRNA) or with a scrambled aspecific siRNA (scr siRNA), incubated for 72 h and then sown on

fibronectin-coated chamber slides. After 24 h, phalloidin staining was performed to visualize cell morphology. Two representative images per group are shown. Original magnification 10×. Scale bars: 100 μm. **b)** The percentage of round cells (compared to spindle migrating cells) present in 10 fields was evaluated and shown in the boxplot. The boxes represent quartiles; the ×, the median; the dots, the individual data points and the whiskers, the variability beyond the upper and lower quartiles. N=3. ***$p < 0.001$ compared to CTR or scr siRNA (Student's t-test).

Supplementary Figure 8. FMRP and Cortactin IF staining in control and FMRP silenced HuH28 cells.

a) The flow chart of Nanostring nCounter analysis after FMRP co-immunoprecipitation (co-IP) experiments is reported. **b)** Untransfected (CTR) or scrambled siRNA transfected (scr siRNA) or *FMR1* siRNAs transfected (*FMR1* siRNA) HuH28 cells were incubated for 72 h and then sown in fibronectin-coated chamber slides. After 24 h an IF was performed. Cells were stained for FMRP (red), Cortactin (green) and DAPI (blue). Representative IF images for the three cellular conditions with matching phase-contrast (PC panels) and merged (MERGE panels) images are shown. Original magnification 20×. Scale bars: 50 μm.

SUPPLEMENTARY MOVIE LEGENDS

Movies 1 and 2. Time-lapse live imaging of HuCCT cells overexpressing FMRP-GFP.

HuCCT cells were transfected with pRRL-h*FMR1*-eGFP lentiviral vector and after 24 h were placed in an epifluorescence microscope for time-lapse live imaging experiments. FITC signal corresponding to FMRP-GFP fusion protein (green) or phase-contrast (PC, grey) were acquired every 10 minutes for 12 h and then 72 total acquisitions were assembled to produce a live imaging movie. Original magnification 20×. Speed, 4 frames per second.

Movies 3. Time-lapse live imaging of HuCCT control cells overexpressing eGFP.

HuCCT cells were transfected with pRRL-eGFP control vector and after 24 h were placed in an epifluorescence microscope for time-lapse live imaging experiments. FITC signal corresponding to eGFP protein (green) or phase-contrast (PC, grey) were acquired every 10 minutes for 12 h and then 72 total acquisitions were assembled to produce a live imaging movie. Original magnification 20×. Speed, 4 frames per second.
